# A burden of rare copy number variants in obsessive-compulsive disorder

**DOI:** 10.1038/s41380-024-02763-7

**Published:** 2024-10-27

**Authors:** Matthew W. Halvorsen, Elles de Schipper, Julia Bäckman, Nora I. Strom, Kristen Hagen, Nordic OCD and Related Disorders Consortium (NORDiC), Matthew W. Halvorsen, Elles de Schipper, Julia Bäckman, Nora I. Strom, Kristen Hagen, Long Long Chen, Diana R. Djurfeldt, Kira D. Höffler, Anna K. Kähler, Paul Lichtenstein, Kathleen Morrill, Hyun Ji Noh, Thorstein Olsen Eide, Tetyana Zayats, Kerstin Lindblad-Toh, Elinor K. Karlsson, John Wallert, Gerd Kvale, Bjarne Hansen, Jan Haavik, Manuel Mattheisen, Christian Rück, David Mataix-Cols, James J. Crowley, Kerstin Lindblad-Toh, Elinor K. Karlsson, Nancy L. Pedersen, John Wallert, Cynthia M. Bulik, Bengt Fundín, Mikael Landén, Gerd Kvale, Bjarne Hansen, Jan Haavik, Manuel Mattheisen, Christian Rück, David Mataix-Cols, James J. Crowley

**Affiliations:** 1 Department of Genetics, University of North Carolina at Chapel Hill, Chapel Hill, NC USA; 2 Centre for Psychiatry Research, Department of Clinical Neuroscience, Karolinska Institutet & Stockholm Health Care Services, Region Stockholm, Sweden; 3 Department of Psychology, Humboldt-Universität zu Berlin, Berlin, Germany; 4 Institute of Psychiatric Phenomics and Genomics (IPPG), University Hospital, LMU Munich, Munich, Germany; 5 Department of Biomedicine, Aarhus University, Aarhus, Denmark; 6 Department of Psychiatry, Molde Hospital, Molde, Norway; 7 Department of Mental Health, Norwegian University of Science and Technology, Trondheim, Norway; 8 Bergen Center for Brain Plasticity, Division of Psychiatry, Haukeland University Hospital, Bergen, Norway; 9 Science for Life Laboratory, Department of Medical Biochemistry and Microbiology, Uppsala University, 751 32 Uppsala, Sweden; 10 Broad Institute of MIT and Harvard, Cambridge, MA 02139 USA; 11 Program in Bioinformatics and Integrative Biology, UMass Chan Medical School, Worcester, MA 01605 USA; 12 Program in Molecular Medicine, UMass Chan Medical School, Worcester, MA 01605 USA; 13 Department of Medical Epidemiology and Biostatistics, Karolinska Institutet, Stockholm, Sweden; 14 Department of Psychiatry, University of North Carolina at Chapel Hill, Chapel Hill, NC USA; 15 Department of Nutrition, University of North Carolina at Chapel Hill, Chapel Hill, NC USA; 16 Institute of Neuroscience and Physiology, University of Gothenburg, Gothenburg, Sweden; 17 Department of Clinical Psychology, University of Bergen, Bergen, Norway; 18 Center for Crisis Psychology, Faculty of Psychology, University of Bergen, Bergen, Norway; 19 Department of Biomedicine, University of Bergen, Bergen, Norway; 20 Dalhousie University, Department of Community Health and Epidemiology & Faculty of Computer Science, Halifax, Nova Scotia Canada; 21 Department of Clinical Sciences, Lund University, Lund, Sweden

**Keywords:** Genetics, Predictive markers

## Abstract

Current genetic research on obsessive-compulsive disorder (OCD) supports contributions to risk specifically from common single nucleotide variants (SNVs), along with rare coding SNVs and small insertion-deletions (indels). The contribution to OCD risk from rare copy number variants (CNVs), however, has not been formally assessed at a similar scale. Here we describe an analysis of rare CNVs called from genotype array data in 2248 deeply phenotyped OCD cases and 3608 unaffected controls from Sweden and Norway. Cases carry an elevated burden of CNVs ≥30 kb in size (OR = 1.12, *P* = 1.77 × 10^−3^). The excess rate of these CNVs in cases versus controls was around 0.07 (95% CI 0.02–0.11, *P* = 2.58 × 10^−3^). This signal was largely driven by CNVs overlapping protein-coding regions (OR = 1.19, *P* = 3.08 × 10^−4^), particularly deletions impacting loss-of-function intolerant genes (pLI >0.995, OR = 4.12, *P* = 2.54 × 10^−5^). We did not identify any specific locus where CNV burden was associated with OCD case status at genome-wide significance, but we noted non-random recurrence of CNV deletions in cases (permutation *P* = 2.60 × 10^−3^). In cases where sufficient clinical data were available (*n* = 1612) we found that carriers of neurodevelopmental duplications were more likely to have comorbid autism (*P* < 0.001), and that carriers of deletions overlapping neurodevelopmental genes had lower treatment response (*P* = 0.02). The results demonstrate a contribution of rare CNVs to OCD risk, and suggest that studies of rare coding variation in OCD would have increased power to identify risk genes if this class of variation were incorporated into formal tests.

## Introduction

Obsessive-compulsive disorder (OCD) is a heritable complex neuropsychiatric condition characterized by persistent, intrusive thoughts and rituals. Current scientific literature supports a genetic contribution to OCD risk. Population-scale epidemiological studies indicate substantial familial clustering of the condition [1–3]. Based on twin study estimates, additive genetic factors account for 47% of variance in obsessive-compulsive symptoms [1]. Analyses of common genetic variation in OCD cases versus controls suggest that common risk variants explain around 28% of the observed phenotypic variance in OCD [4].

Rare variant studies of OCD published in recent years have primarily involved whole exome sequencing (WES) of trio cohorts (consisting of parents and an affected proband). Cappi et al. first described an analysis of WES data for 184 OCD trios and detected an excess of de novo (variant found in the proband, but absent in both parents) damaging coding single nucleotide variants (SNVs) and insertion-deletions (indels) in probands relative to unaffected trios, along with two genes recurrently hit with damaging mutations, *CHD8* and *SCUBE1* [5]. More recently, Halvorsen et al. described an analysis of 1313 OCD cases, of which 587 were probands in complete trios [6]. In accordance with Cappi et al., authors noted an excess of de novo damaging coding SNVs and indels in these OCD trios relative to unaffected trios. They also observed a general excess of protein-truncating SNVs and indels in singleton cases relative to ancestry-matched controls. They identified *CHD8* as a probable risk gene (Q < 0.^3^) in their analyses, which utilized summary statistics from Cappi et al., and identified an additional damaging coding de novo variant in this gene within their newly published trios [6]. It is critical to note that neither of these studies featured any assessment of copy number variants (CNVs). Since these variants are characterized by the deletion or duplication of thousands of bases, it is reasonable to hypothesize that in a sufficiently powered case/control comparison, given the excess of protein-truncating SNVs and indels already seen in OCD, there might be a similar excess of CNVs in OCD cases specifically impacting protein-coding genes.

Several genome-wide CNV studies of OCD have been published over the years (Supplementary Table 1), but they either have smaller sample sizes, or do not specifically focus on OCD as a phenotype. The largest CNV study of OCD is from McGrath et al. in 2014, as part of a joint study of OCD and Tourette Syndrome (TS) cases [7]. The study did not describe a general excess of rare CNVs, but did note that cases had an elevated rate of neurodevelopmental deletions relative to controls that was not statistically significant [7]. There have been other more recent genome-wide CNV studies of OCD, but none featuring an exhaustive case/control comparison of CNV burden at a similar scale of McGrath et al. or the exome study described in Halvorsen et al. [7–11].

Given the excess of protein-truncating SNVs and indels already seen in OCD WES studies, we hypothesized that there might be a similar excess of CNVs in OCD cases, and that these CNVs specifically impact protein-coding genes. To test this hypothesis, we designed a case/control CNV study which benefits from usage of a more recently-developed genotype array platform (the Illumina Global Screening Array series; GSA), an ancestrally homogenous Scandinavian population ideal for genetic study, and rich clinical data available for enrolled cases.

## Methods

### Samples

All OCD cases included in this study were collected in Sweden and Norway as part of the Nordic OCD and Related Disorders Consortium (NORDiC). The rationale, design and methods of the NORDiC study have been described previously [12]. In Sweden, the case-control arm of the study is referred to as NORDiC-SWE and all samples were collected across Sweden between 2015 and 2019. This study was approved by a local ethics board (Stockholm Regional EPN) and all participants provided informed consent. NORDiC-SWE OCD cases (63% female) have a primary International Classification of Disease, 10th revision [13] and/or Diagnostic and Statistical Manual of Mental Disorders, Fifth Edition [14] diagnosis of OCD from a multidisciplinary specialist OCD team established with a semi-structured instrument such as the Mini-International Neuropsychiatric Interview [15] or the Structured Clinical Interview for DSM Disorders [16]. All patients were included in the study regardless of psychiatric comorbidity, as long as they fulfilled strict diagnostic criteria for OCD. Patients were excluded in cases of diagnostic uncertainty, such as OCD secondary to a neurological disorder or CNS insult, or where the differential diagnosis between OCD and an alternative condition was unclear. Swedish controls were sampled from LifeGene [17], a prospective population-based cohort of around 50,000 individuals in Sweden. Controls were unrelated to any OCD case to the third degree and unaffected with OCD based on self-report. Since the controls were inherited from those used for a GWAS of anorexia nervosa [18], potential controls were excluded if they had a lifetime history of anorexia nervosa and were largely female (~91%). Participants provided either blood or saliva for DNA extraction.

In Norway, the case-control arm of the study is referred to as NORDiC-NOR and all samples were collected across Norway between 2016 and 2019. This study was approved by the regional ethics board (REK West) and all participants provided informed consent. NORDiC-NOR OCD cases (65% female) had the same diagnostic process, and the inclusion and exclusion criteria as those in Sweden. Norwegian controls (50% female) were selected from NORMENT and were ages 18–65 at time of collection. They were screened for psychiatric illness via questionnaires, and included individuals have indicated that neither they nor any first-degree relatives have undergone any formal treatment. Participants provided either blood or saliva for DNA extraction. See [19] for more details.

### Array genotyping

The majority of samples in this study were genotyped on the Illumina GSA version 1, 2 or 3 (GSAv1, v2, v3), which include a common core of ~600,000 SNPs [20]. The one exception was the Norwegian controls, which were genotyped at deCODE Genetics using any array derived from GSAv1 that contained the same common core of SNPs [19]. The GSA samples were genotyped at LIFE&BRAIN in Bonn, Germany and the Norwegian controls were genotyped at deCODE Genetics in Reykjavík, Iceland. Some cohorts were genotyped in multiple waves and Supplementary Table 2 provides the number of samples for each wave.

### Processing genotype array data

We obtained raw genotype array data (IDATs) for cases and controls, and processed them using the gtc2vcf pipeline (https://github.com/freeseek/gtc2vcf). We transformed IDATs into dataset-level variant call format (VCF) files with reference alleles listed relative to human reference build 37 (see Supplementary Methods for details), which simplified the dataset merge process. We merged all 10 input datasets on a subset of variants that have genotype missingness <0.02 in each individual dataset. We identified a total of 542,466 variants that fit the full set of criteria. As a precaution, we took the subset of 537,278 variants that were non-ambiguous (not A/T or G/C), and were not indels for the merger. The raw merged dataset consisted of genotypes across these 537,278 variants for 2885 cases and 4227 controls.

### Quality Control (QC)

We carried out several rounds of sample-level QC using PLINK v1.90b4.9 to ensure that any case/control association results were not influenced by poor sample quality, sample swapping, cryptic relatedness or ancestry differences. See Supplementary Methods for a description of the full procedure, and the number of samples removed at each step. After sample-level QC we were left with a total of 2325 cases and 3790 controls suitable for inclusion in a comparison of CNV burden between OCD cases and controls.

### CNV calling

We called CNVs on all NORDiC OCD cases and Swedish and Norwegian controls which we had sample-level Log R ratio (LRR) and B allele frequency (BAF) data for. All CNV calls were made on the same set of 537,278 variants common to all data described previously. The calling procedure utilized PennCNV v1.0.5 and QuantiSNP v2.2 to generate separate CNV callsets for each single sample, and then defined CNV loci based on the intersection of these callsets. See the Supplemental Methods section for a description of the full procedure.

### Sample-level QC specific to intensity metrics

Within each individual dataset, we performed outlier pruning on sample-level intensity metrics to remove poor-quality samples likely to have aberrant CNV call metrics. All metrics were computed by the PennCNV command ‘detect_cnv.pl’. Our dataset-level outlier pruning was focused on the standard deviation of the Log R Ratio (LRRSD), absolute value of waviness factor (absWF) and BAF drift. For each metric in a given dataset, a sample was marked as an outlier if it fell beyond 3 standard deviations (SDs) of the mean. A sample was removed if it was an outlier for any of these metrics. After dataset-level pruning, all remaining samples had LRRSD ≤ 0.2, absWF ≤ 0.02, and BAF drift ≤ 0.001.

We next produced density plots of total CNV count per sample and total number of bases occupied by CNV per sample, and noted a small number of samples with unusually high counts that dataset-level QC failed to exclude. Based on visual inspection of the kernel density plots for the sample-level raw CNV counts and the number of bases occupied by raw CNVs, we removed samples that had over 20MB of basepairs occupied by raw CNV calls, or a total number of separate raw CNV calls greater than 20.

### CNV filtering

The qualifying (≥15 probes, ≥30 kb) CNV callset was put through a series of filter steps using code adapted from [21] in order to produce a set of analysis-ready calls that are rare, and do not overlap loci naturally prone to copy number alterations. First, we removed CNVs that overlapped (here defined as >30%) with loci 500 kb from telomeres, or 500 kb away from designated centromere loci. Next, we removed CNVs with 30% of bases overlapping “non-defined” (i.e., polyN) portions of the GRCh37 reference. We also removed CNVs overlapping previously reported and described segmental duplication loci [22]. Next, we removed CNVs where >30% of bases overlapped loci from Repeatmasker (http://www.repeatmasker.org), corresponding to simple repeat, low complexity or satellite loci. CNVs that overlapped gene regions for T cell receptors or immunoglobulins were removed next. We removed loci where CNV calls were associated with samples from Epstein Barr Virus-transformed Lymphoblastoid Cell Lines [23], as utilized in Huang et al. [24]. Next, calls were subjected to a series of filters on CNV frequency. Calls were required to be found at <1% frequency in gnomAD v2.1 non-neuro global and subpopulations [25]. In addition, calls were required to be found at <1% frequency in the full combined case/control dataset, along with <1% frequency in each additional input dataset. The final step of our CNV filtering protocol utilized marker-level BAF and LRR values in a callsite for each given carrier sample, and used BAF metrics to validate or reject a call by determining if the distribution of BAF values is consistent with reported copy state, as described in https://biopsych.dk/iPsychCNV/ and utilized in [26] (see Supplemental Methods).

### Gene-based and breakpoint-based association tests

We used gene-based and breakpoint-based tests to determine if there were single genes or loci where overlapping CNVs were associated with OCD case status to a degree surviving multiple test correction, while also examining evidence of test statistic inflation. We defined four separate case/control groups for this analysis : 1) Swedish male, 2) Swedish female, 3) Norwegian male, and 4) Norwegian female. For each locus, we compared the proportion of cases with at least one rare, overlapping CNV to the proportion in controls, using a two-sided Cochran–Mantel–Haenszel exact test. To estimate genomic inflation, we used a case/control permutation-based approach described in ref. [6]. Before formal tests we tested for genomic inflation of non-overlapping locus-based test statistics, and excluded CNVs overlapping 3 loci as a result (see Supplemental Methods).

We conducted gene-based tests of CNV burden in a manner described previously by others [27]. We tested deletions and duplications separately, and merged neighboring genes into single units if over 50% of overlapping CNVs impact both genes [27], leading to 988 separate tests in total. We used the Benjamini–Hochberg procedure to control for false discovery, defining results where false discovery rate (FDR) < 0.05 as significant.

We also constructed association tests based on CNV breakpoints. As with gene-based tests, we merged probes into separate units based on whether they are shared by over 50% of CNVs that impact them. We also used the same *p*-value adjustment procedure here as before. Once again, test statistics are well controlled for deletions (lambda = 1.02) and for duplications (lambda = 1.01).

### Power analysis for association tests

We conducted power analyses to produce estimates for the types of potential risk CNVs for OCD we might have reasonable power to implicate, given a prevalence estimate of 0.01, a sample size of 2248 cases and 3608 controls and multiple testing correction for 20,000 genes. We saw that in power calculations we had 80% power to detect risk CNVs with relative risk and corresponding control frequencies ranging from 69.8 at a frequency of 0.0001 to a relative risk at 2.5 at a frequency of 0.01. When comparing our power curves to risk CNVs identified from a well-powered case/control CNV study of Schizophrenia [27], we see that we are largely underpowered to implicate similar CNVs in our study. We produced power curves for a case/control comparison at the same sample size as described in the Schizophrenia CNV study [27] and we see that in this theoretical study design we should have sufficient power to detect these OCD risk CNVs should they exist. See Supplemental Methods section for an in-depth discussion of the power analysis and plotting of power curves.

### Global CNV burden tests

We tested for an association between case status and global CNV burden using linear and logistic regression models, with covariates. We first constructed a null model for the total count of raw CNVs per sample. Without covariates, we saw that case status was associated with the raw number of CNV calls made in a given sample (estimate = −0.17, *P* = 0.04). We added to this model major principal components representative of ancestry (PCs 1–5) and sex. Notably, PC5 is a clear predictor of Swedish versus Norwegian ancestry, in a manner that is not dataset-specific (see Supplementary Figs. 2 and 3). We considered additional covariates (PCs 6–20, LRRSD) and added them to the model if they were associated with both raw CNV count and with case status. We found PC 7 and LRRSD to be associated with both raw CNV count and case status, and added them to the model as well. The logistic regression model in global comparisons of burden was:

*OCD_case* ~ *PC1* + *PC2* + *PC3* + *PC4* + *PC5* + *PC7* + *SEX* + *LRR_SD* *+* *burden_metric*

A linear regression model used these same covariates, but here instead the so-called ‘burden metric’ was the outcome and OCD case status was the critical predictor. All *p*-values reported from these tests are two-sided.

## Results

### Case/control cohort

**Fig. 1:**
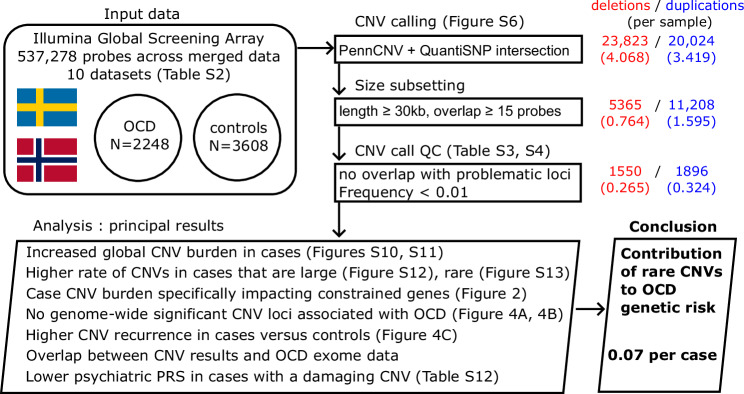
An overview of the study design and principal results from the analyses carried out. We have constructed a CNV case/control study using samples from Illumina GSA and its derivatives. We use extensive CNV call quality control to bring our CNV call rate down to around 0.6 per sample. Our principal results all point to a contribution to OCD genetic risk from CNVs that are at least 30 kb in size and a frequency <0.01, at a rate per sample of around 0.07 (95% CI 0.02–0.11, *P* = 2.58 × 10^−3^).

The raw case/control cohort consisted of 2885 cases and 4227 controls, spread across 10 datasets (Fig. 1, Supplementary Table 2). All samples were genotyped on versions or derivatives of the Illumina GSA (Supplementary Table 2). We found a total of 537,278 probes shared across all datasets within this study, and utilized these probes for both quality control and CNV calling.

We isolated a subset of the cohort that were of high technical quality and were suitable for case/control comparisons (see Methods and Supplemental Methods for details). A total of 2248 cases and 3608 controls were included in our formal CNV analyses (Fig. 1, Supplementary Table 2). We did not see evidence of stratification of OCD case status across any of the major principal components (Supplementary Fig. 1), though it was clear that Swedish and Norwegian ancestry did separate across these components and should be accounted for (Supplementary Figs. 2 and 3). Most of the variance within the data was explained by the first 5 PCs (Supplementary Fig. 4).

### CNV calling and filtering

We called and analyzed CNVs at least 30 kb in size and spanning at least 15 probes. A large number of these calls were present in sample-level data (1.42 calls per sample, Supplementary Tables 3–5). We retained CNVs outside of genomic loci prone to noisy intensity values, and were found at a frequency <0.01 in the cohort as well as the gnomAD v2.1 structural variant callset (see Methods). This procedure led to a higher degree of comparability across separate datasets (0.59 calls per sample, Supplementary Tables 3–5).

We compared cases and controls for evidence of systemic differences in raw CNV call count, LRRSD and filtered CNV call count. LRRSD metrics across datasets indicated that data were of good quality. Looking at ANGI controls, which underwent clustering before we received the data, the mean LRRSD metrics were higher, but still in range of other included datasets (Supplementary Fig. 5). As indicated before, raw CNV call counts had appreciable differences between datasets, while QC-pass CNV call counts were well-harmonized across the data (Supplementary Figs. 6 and 7).

Out of an abundance of caution, we compared the global burden of the smallest size bin of CNVs assessed in cases versus controls (30–100 kb) to determine if there are clusters of calls that pile up in a manner suggestive of batch effect. We specifically noted an elevation of CNV deletion signal (lambda = 1.21), which was driven exclusively by 19 calls clustering around 3 loci (see Methods). We excluded these CNVs from further analyses, eliminating genomic inflation for deletions within this size bin (lambda = 0.98, Supplementary Fig. 8). No similar clustering that was suggestive of batch effect was present in CNVs calls between 100 kb and 500 kb in size (Supplementary Fig. 9).

### Global CNV burden

We found that OCD cases had an excess burden of rare CNVs at least 30 kb in size relative to unaffected controls (OR = 1.12, *P* = 1.77 × 10^−3^). More of this excess burden appears to come from deletions (OR = 1.16, *P* = 8.41 × 10^−3^) than from duplications (OR = 1.09, *P* = 0.06, Supplementary Fig. 10). Leave-one-out analyses showed that these results were not driven by any lone input dataset (Supplementary Fig. 10), or by one covariate (Supplementary Fig. 11). Every additional basepair of deletion made a sample more likely to be a case (OR = 1.047 per 100 kb, *P* = 2.31 × 10^−3^), along with every additional basepair of duplication (OR = 1.033 per 100 kb, *P* = 1.81 × 10^−3^). Consistent with this, OCD cases carried an excess burden of large (>1MB) CNVs (OR = 2.01, *P* = 3.35 × 10^−4^, Supplementary Fig. 12). Ultrarare CNVs observed only once in the case/control cohort conferred greater relative risk for OCD (OR = 1.21, *P* = 1.30 × 10^−3^, Supplementary Fig. 13), consistent with particularly penetrant, risk-conferring CNVs being subject to negative selection.

### CNV burden is concentrated in protein-coding regions

OCD cases were more likely to carry CNVs that impact protein-coding regions of the genome (OR = 1.19, *P* = 3.07 × 10^−4^). There was no evidence for a case burden relative to controls for CNVs not overlapping any protein-coding bases (OR = 1.04, *P* = 0.50). Consistent with the burden of CNVs in cases, the accumulation of CNV-impacted protein-coding genes increased OCD case risk (OR = 1.07 and *P* = 1.99 × 10^−3^ for each deletion-impacted gene, OR = 1.04 and *P* = 3.48 × 10^−3^ for each duplication-impacted gene).

**Fig. 2:**
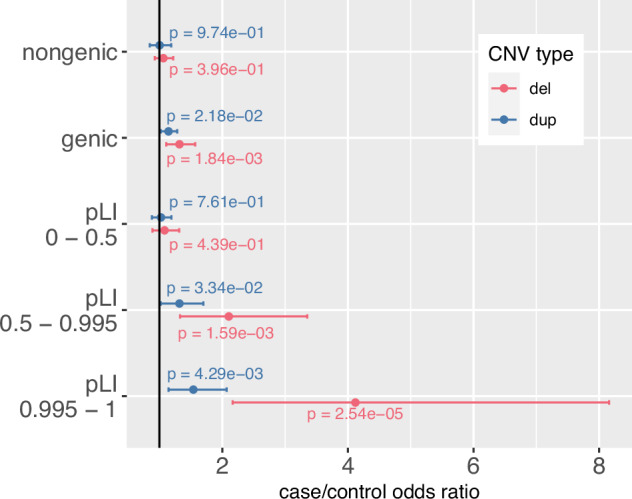
CNV burden (deletions, duplications) partitioned by overlap with protein-coding genes. The odds ratio estimate for case status for each additional CNV (deletions in red, duplications in blue) is depicted with a dot while the 95% confidence interval for the estimate is depicted with bars. Unadjusted *p*-values are provided for each test result. There is no evidence for case risk being conferred by CNVs that don’t overlap a protein-coding base (*P* > 0.05 for both deletions and duplications). CNVs that confer OCD risk instead appear to overlap protein-coding regions, specifically those that code for genes that are loss-of-function intolerant (pLI > 0.5).

Case CNV signal was concentrated within genes that are dosage sensitive. Cases carried an excess of CNVs that overlap at least one protein-coding gene that is more likely to be intolerant to loss-of-function (pLI >0.5, OR = 1.60, *P* = 6.37 × 10^−8^, Fig. 2) [28]. There was no difference in burden of CNVs that do not carry at least one of these genes (OR = 1.04, *P* = 0.48). We also utilized more recently described [21] sets of data-derived haplosensitive and triplosensitive genes and found that CNV burden was elevated primarily within haplosensitive genes (Supplementary Fig. 14). Finally, we carried out a series of tests to determine if case sample country-of-origin or sex influence deleterious CNV burden (see Supplementary Methods). We failed to detect a difference in deleterious CNV burden between Swedish and Norwegian cases (Supplementary Fig. 15) and between male and female cases (Supplementary Fig. 16).

### CNV burden impacting evolutionarily constrained bases

**Fig. 3:**
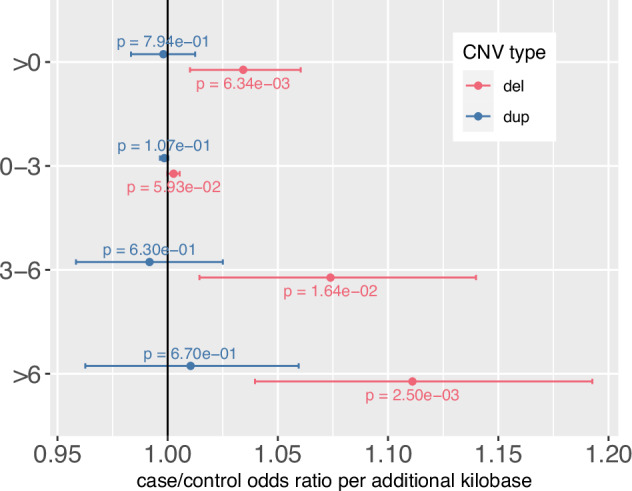
Number of bases impacted by CNVs (deletions, duplications) partitioned by mammalian constraint score. The odds ratio estimate for case status for each additional kilobase impacted by CNVs (deletions in red, duplications in blue) is depicted with a dot while the 95% confidence interval for the estimate is depicted with bars. Unadjusted p-values are again provided for each test result. In general, each kilobase of DNA that is deleted increases OCD risk, in a manner where the risk conferred increases when the bases deleted are more constrained. This effect is not observed for duplications.

OCD cases had a higher number of evolutionarily constrained bases impacted by CNV deletions than controls (OR = 1.03 per kbp, *P* = 6.34 × 10^−3^). There was no significant case/control difference in the number of constrained bases impacted by duplications (OR = 0.998 per kbp, *P* = 0.79). We found that CNV deletion burden preferentially loads onto bases with particularly high phyloP scores (Fig. 3), consistent with deletions impacting genomic loci that are intolerant to variation. Repeating this test on CNVs that did not impact a coding base, we did not note any significant case/control difference in constrained bases burdened by CNVs (Supplementary Fig. 17).

### Gene-based tests of CNV burden

**Fig. 4:**
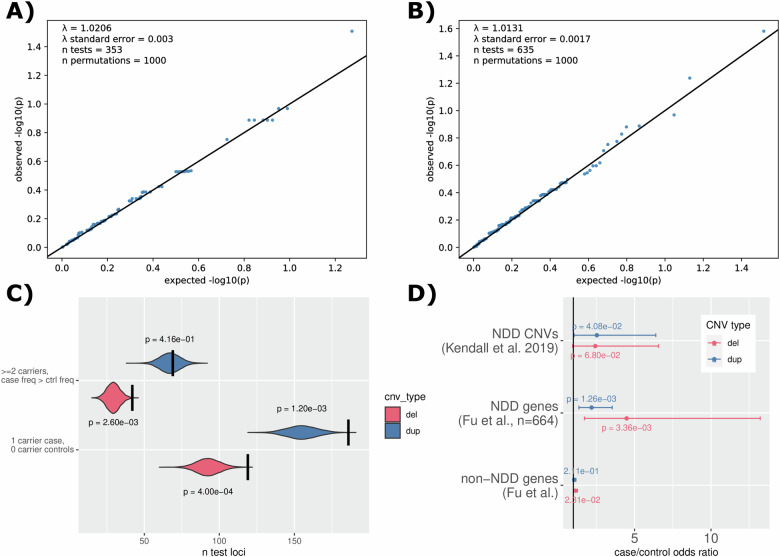
Lack of genome-significant CNV impacted loci in OCD cases versus controls likely due to low power. **A** QQ plot for clumped gene-based test results specific for deletions. **B** QQ plot for clumped gene-based test results specific for duplications. **C** Results from permutation tests of CNV burden in recurrence in OCD cases relative to controls. **D** Tests of association between neurodevelopmental disorder (NDD) CNV burden and OCD case status. Odds ratio for each additional CNV (deletions in red, duplications in blue) is depicted with a dot while the 95% confidence interval for the estimate is depicted with bars. Unadjusted *p*-values are also provided. All detectable duplication excess in cases appears to impact neurodevelopmental genes (*n* = 664, from Fu et al. [30]) while the deletion excess in cases appears to impact both neurodevelopmental genes and unknown genes outside of this geneset.

We failed to identify any test statistics where the level of significance passed the threshold for significance (988 tests, FDR-adjusted *P* < 0.05). There was no evidence of genomic inflation within deletion test statistics or duplication test statistics (lambda = 1.02 and lambda = 1.01, respectively, Fig. 4A, B). In spite of no individual loci being implicated, the overall CNV burden described in OCD cases suggests that a larger cohort size is likely to provide the sufficient power required. In particular, cases were more likely to have CNVs where only one sample overlaps the affected area, and specifically, cases have an elevation of loci impacted by at least two deletions beyond what case/control permutation predicts (Fig. 4C). Summary statistics from these tests have been included (Supplementary Table 7), along with statistics from breakpoint-based tests (Supplementary Table 8).

### Burden of neurodevelopmental CNVs

In general, we found that OCD cases carried a higher burden of neurodevelopmental CNVs than controls. Burden of neurodevelopmental CNVs as defined in Kendall et al. [29] increased OCD case risk (OR = 2.49, *P* = 6.04 × 10^−3^), as did burden within specific genes implicated with neurodevelopmental disorders from Fu et al. [30] (*n* = 664, OR = 2.54, *P* = 1.91 × 10^−5^). Although the deletion contribution to this result was higher, there was a discernible contribution from duplications as well (Fig. 4D).

### Pathway enrichment analyses

We ran a series of pathway enrichment analyses (see Supplemental Methods for details) on genesets derived from 2187 Gene Ontology (GO) terms and 37 tissues from GTEx (13 from the brain) using files generated via Bryois et al. [31]. We included genes with pLI >0.5 (4543 genes) as a positive control given the enrichment described earlier, and a set of tolerant genes (pNull >0.5, 4540 genes) used as a negative control. Out of 2226 total tests, only one test passed the FDR-corrected significance threshold of 0.1, the positive control pLI >0.5 (Supplementary Table 9). We attempted to use this signal to add additional power to our pathway enrichment analyses by specifically focusing on geneset tests subsetted specifically on genes with pLI >0.5, controlling for the total of intolerant genes impacted per person, and found that across these tests (795 total), a total of 7 tests survived FDR correction (Supplementary Table 10). Of these tests, 5 were expression profiles from GTEx brain tissue, an event unlikely by chance given only 13 brain tissues tested total (two-sided fisher’s exact test *P* = 9 × 10^−9^). Significant brain tissues include the Substantia nigra, Hippocampus and the Hypothalamus.

### Overlap with exome sequencing studies of OCD

We found non-random overlap between genes impacted by case-only single-gene CNVs in our study and prior OCD exome study statistics from Supplementary Table S15 of ref. [6]. We derived a set of genes from our analyses that were impacted by at least one single-gene case CNV and no single-gene control CNV (*n* = 149 genes). These genes had an elevated count of loss-of-function and damaging missense de novo mutations across 771 trios (observed = 9, expected = 3.94, one-sided poisson *P* = 0.02) and an elevated count of loss-of-function variants in 476 cases versus 1761 controls (observed = 26, expected = 17.30, one-sided poisson *P* = 0.03). We set up a Transmission and De Novo Association (TADA) analysis using the same methods described previously [6] and the summary statistics from Supplementary Table S15, with NORDiC case/control count statistics added. No genes beyond the already-described *CHD8* passed the threshold of Q < 0.3 for being classified as a probable risk gene (Supplementary Table 11), though the gene that comes closest, *ZMYM2* (Q = 0.32), has been implicated in neuropsychiatric phenotypes across multiple publications [30, 32, 33].

### OCD polygenic risk in deleterious CNV carriers

We hypothesized that individuals carrying deleterious (pLI >0.995, neurodevelopmental as in Kendall et al., or neurodevelopmental as in Fu et al.) CNVs were more likely to have lower neuropsychiatric polygenic risk. This would be consistent with higher-powered studies of other neuropsychiatric conditions [34]. To test this, we utilized polygenic risk scores (PRS) computed from three different GWAS summary statistics: standing height (Pan-UKB, https://pan.ukbb.broadinstitute.org) (*N* = 360,388, as a negative control), OCD [4] (2688 cases, 7037 controls), and a cross-disorder study of psychiatric conditions [35] (162,151 cases, 276,846 controls). We tested for an association between deleterious CNV burden and normalized PRS, using the same covariates as those in global CNV burden analyses, and performing separate tests for deletions and duplications.

Of the six tests we performed (Supplementary Table 12), we identified one significant (*p* < 0.05) association, between deleterious CNV deletions and cross-psychiatric condition study PRS. In this comparison, deleterious CNV deletion carriers in our case cohort had lower normalized psychiatric PRS than non-carriers (estimate = −0.45, *P* = 3.35 × 10^−3^). While this PRS is not OCD-specific, the summary statistics underlying it do include OCD cases, and given how much larger the sample size is, it likely captures pleiotropic common risk variants that increase risk for multiple psychiatric conditions at once.

### Clinical features of carriers of deleterious CNVs

We performed an analysis of clinical features of case carriers of these deleterious CNVs versus non-carrier cases (see Supplementary Table 13 for carrier status per sample). We focused on the Swedish subset of the case cohort (*n* = 1612) where we had access to detailed clinical information on each participant. Details of the cohort characteristics, treatments and outcome measures are described in detail in the study protocol [12]. Briefly, participants were recruited through a network of specialist OCD clinics that have highly standardized assessment and treatment protocols or via self-referral to a dedicated study website. All diagnoses were confirmed through a structured diagnostic interview. For those receiving treatment, this consisted of specialized cognitive-behavior therapy and/or serotonin reuptake inhibitors delivered by highly experienced teams. The primary outcome measure was the clinician-rated Yale-Brown Obsessive Compulsive Scale (YBOCS; score range 0–40, with higher scores denoting more severe symptoms).

We first explored the association between deleterious deletions and duplications and the presence of key psychiatric comorbidities (ASD, ADHD, TS/chronic tic disorder, schizophrenia, bipolar disorder and eating disorders) through contingency tables and Chi-Square statistics (or Fisher exact tests, when relevant). We found that 6 (4.1%) of the 147 individuals with comorbid ASD had neurodevelopmental duplications, compared to 6 (0.4%) of 1465 individuals without comorbid ASD (Chi-square = 24.3, df = 1, *P* < 0.001). The remaining psychiatric disorders were not significantly associated with neurodevelopmental duplications. No significant associations emerged for neurodevelopmental deletions. We also examined if neurodevelopmental duplications and deletions were associated with reported age of symptom onset or pretreatment YBOCS scores, but they were not (*P* values < 0.05).

We further explored if the presence of duplications or deletions was associated with treatment outcomes in a sub-cohort of Swedish individuals with complete treatment data (*n* = 846). We found that individuals with deletions (but not duplications) in specific neurodevelopmental disorder genes improved on average 16% on the YBOCS, whereas individuals without such deletions improved 47% on the YBOCS, a statistically significant difference (independent samples *t*-test; *t* = −3.03, df = 854, 2-sided *P* = 0.02).

## Discussion

We have compiled what, to our knowledge, is currently the largest OCD case/control study of rare CNVs, and our results support a contribution of these variants to OCD genetic risk. This contribution came specifically from rare CNVs that overlap protein-coding regions of the genome, as there was no detectable difference in noncoding CNV burden between cases and controls. Large, ultra-rare CNVs appeared to confer the highest amount of OCD relative risk. Even when controlling for the total number of CNV-impacted bases, OCD cases had a higher number of deleted bases that are under high mammalian evolutionary constraint. In a manner consistent with OCD WES studies, coding region CNVs impacting loss-of-function intolerant protein-coding genes appear to confer more substantial OCD risk than those that do not. There was no single locus in the genome where CNV burden predicted OCD case status at a level that survived multiple test correction. The distribution of case CNV calls in the genome was non-random, and consistent with a pattern in which distinct CNV risk loci exist, but we have insufficient sample size to be able to detect them.

Our study benefited from the uniquely rich clinical information that is available from the participants in the NORDiC study. In particular, we established a specific association between neurodevelopmental duplications and ASD (not other comorbidities), although no significant associations emerged for neurodevelopmental deletions. The results suggest that whereas neurodevelopmental duplications in OCD can be, at least in part, explained by the presence of comorbid ASD, our findings regarding deletions appear to be independent of key psychiatric comorbidities. We also found a tentative association between deletions and multimodal treatment response in a sub-cohort of individuals who had treatment outcome data, whereby individuals with deletions in neurodevelopmental disorder genes were less likely to respond to treatment. However, these results should be interpreted with caution because this analysis only included 8 cases with deletions in neurodevelopmental disorder genes. Larger samples are needed to confirm this finding.

An attribute of our study that could be interpreted as both a strength and a weakness is the ancestral homogeneity of the cohort. While this led to a cleaner analysis that is unlikely to be influenced by substantial differences in ancestry, it does not address European bias and subsequent inequity present in most genetic studies [36]. We note that the effects of negative selection mean that results here are likely generalizable across ancestries. OCD cases here are enriched for CNVs overlapping protein-coding genes which are depleted of damaging variation across multiple ancestries [28]. Because of this, these variants are likely subject to negative selection, irrespective of ancestry. Consistent with this, a recent cross-ancestry analysis of Schizophrenia cases and unaffected controls [37] saw significant overlap with rare variant burden in constrained genes already highlighted in the SCHEMA study [33].

The case/control CNV callset here and the previously published gene-based WES summary statistics from Halvorsen et al. 2021 overlap in a nonrandom manner consistent with the presence of multiple OCD risk genes impacted in both datasets [6]. This indicates that the process of calling CNVs from WES data and forming gene-based summary statistics that incorporate SNV, indel and CNV calls is a worthwhile endeavor. Consistent with this, recent large WES analyses have benefited greatly from incorporating CNV call information into gene based tests, and methods for making CNV calls from WES data and incorporating them into analyses have been optimized [30, 38]. CNV burden and damaging SNV/indel burden, in a scenario where sample size is sufficiently large, should point to a consistent core set of risk genes where damaging coding variation substantially increases risk of OCD.

## Supplementary information

Supplementary Information

Supplemental Methods

Supplemental Tables

Analysis code

## Data Availability

Genome-wide locus-based case/control test statistics have been provided in Supplementary Tables 7 and 8. Call information for deleterious CNVs detected in OCD cases can be found in Supplementary Table 13. Raw data from OCD cases will be made available for protected access in a manner compliant with European Union General Data Protection Regulations.

## Code availability

Initial processing of raw genotype array data using the gtc2vcf pipeline (https://github.com/freeseek/gtc2vcf). Processing of PLINK files, along with relatedness checks and PCA, were carried out using PLINK v1.90b4.9 (https://www.cog-genomics.org/plink/). CNV calling was carried out using PennCNV (https://penncnv.openbioinformatics.org/) and QuantiSNP (https://github.com/cwcyau/quantisnp). Filtering of CNVs was done using code adapted from https://github.com/talkowski-lab/rCNV2. The code we wrote and utilized for analysis of processed data and production of manuscript figures have been provided as a supplemental file.

## References

[1] Mataix-Cols D, Boman M, Monzani B, Rück C, Serlachius E, Långström N, et al. Population-based, multigenerational family clustering study of obsessive-compulsive disorder. JAMA Psychiatry. 2013;70:709–17. 23699935 10.1001/jamapsychiatry.2013.3

[2] Browne HA, Hansen SN, Buxbaum JD, Gair SL, Nissen JB, Nikolajsen KH, et al. Familial clustering of tic disorders and obsessive-compulsive disorder. JAMA Psychiatry. 2015;72:359–66. 25692669 10.1001/jamapsychiatry.2014.2656

[3] Blanco-Vieira T, Radua J, Marcelino L, Bloch M, Mataix-Cols D, do Rosário MC. The genetic epidemiology of obsessive-compulsive disorder: a systematic review and meta-analysis. Transl Psychiatry. 2023;13:230. 37380645 10.1038/s41398-023-02433-2 PMC10307810

[4] International Obsessive Compulsive Disorder Foundation Genetics Collaborative (IOCDF-GC) and OCD Collaborative Genetics Association Studies (OCGAS). Revealing the complex genetic architecture of obsessive-compulsive disorder using meta-analysis. Mol Psychiatry. 2018;23:1181–8. 28761083 10.1038/mp.2017.154 PMC6660151

[5] Cappi C, Oliphant ME, Péter Z, Zai G, Conceição do Rosário M, Sullivan CAW, et al. De Novo Damaging DNA Coding Mutations Are Associated With Obsessive-Compulsive Disorder and Overlap With Tourette’s Disorder and Autism. Biol Psychiatry. 2020;87:1035–44. 31771860 10.1016/j.biopsych.2019.09.029 PMC7160031

[6] Halvorsen M, Samuels J, Wang Y, Greenberg BD, Fyer AJ, McCracken JT, et al. Exome sequencing in obsessive-compulsive disorder reveals a burden of rare damaging coding variants. Nat Neurosci. 2021;24:1071–6. 34183866 10.1038/s41593-021-00876-8

[7] McGrath LM, Yu D, Marshall C, Davis LK, Thiruvahindrapuram B, Li B, et al. Copy number variation in obsessive-compulsive disorder and tourette syndrome: a cross-disorder study. J Am Acad Child Adolesc Psychiatry. 2014;53:910–9. 25062598 10.1016/j.jaac.2014.04.022 PMC4218748

[8] Gazzellone MJ, Zarrei M, Burton CL, Walker S, Uddin M, Shaheen SM, et al. Uncovering obsessive-compulsive disorder risk genes in a pediatric cohort by high-resolution analysis of copy number variation. J Neurodev Disord. 2016;8:36. 27777633 10.1186/s11689-016-9170-9 PMC5070001

[9] Zarrei M, Burton CL, Engchuan W, Young EJ, Higginbotham EJ, MacDonald JR, et al. A large data resource of genomic copy number variation across neurodevelopmental disorders. NPJ Genom Med. 2019;4:26. 31602316 10.1038/s41525-019-0098-3 PMC6779875

[10] Mahjani B, Birnbaum R, Buxbaum Grice A, Cappi C, Jung S, Avila MN, et al. Phenotypic Impact of Rare Potentially Damaging Copy Number Variation in Obsessive-Compulsive Disorder and Chronic Tic Disorders. Genes. 2022;13:1796. 10.3390/genes13101796 PMC9601402 36292681

[11] Grünblatt E, Oneda B, Ekici AB, Ball J, Geissler J, Uebe S, et al. High resolution chromosomal microarray analysis in paediatric obsessive-compulsive disorder. BMC Med Genomics. 2017;10:68. 29179725 10.1186/s12920-017-0299-5 PMC5704537

[12] Mataix-Cols D, Hansen B, Mattheisen M, Karlsson EK, Addington AM, Boberg J, et al. Nordic OCD & Related Disorders Consortium: Rationale, design, and methods. Am J Med Genet B Neuropsychiatr Genet. 2020;183:38–50. 31424634 10.1002/ajmg.b.32756 PMC6898732

[13] Organisation mondiale de la santé, World Health Organization, WHO, WHO Staff. The ICD-10 Classification of Mental and Behavioural Disorders: Clinical Descriptions and Diagnostic Guidelines. Geneva, Switzerland: World Health Organization; 1992.

[14] Diagnostic and Statistical Manual of Mental Disorders: DSM-5. Arlington, VA, USA: American Psychiatric Association; 2013.

[15] Sheehan DV, Lecrubier Y, Sheehan KH, Amorim P, Janavs J, Weiller E, et al. The Mini-International Neuropsychiatric Interview (M.I.N.I.): the development and validation of a structured diagnostic psychiatric interview for DSM-IV and ICD-10. J Clin Psychiatry. 1998;59:22–33. 9881538

[16] First MB, Williams JBW, Karg RS, Spitzer RL. SCID-5-CV: Structured Clinical Interview for DSM-5 Disorders : Clinician Version. Arlington, VA, USA: American Psychiatric Pub; 2015.

[17] Almqvist C, Adami H-O, Franks PW, Groop L, Ingelsson E, Kere J, et al. LifeGene-a large prospective population-based study of global relevance. Eur J Epidemiol. 2011;26:67–77. 21104112 10.1007/s10654-010-9521-x PMC7087900

[18] Watson HJ, Yilmaz Z, Thornton LM, Hübel C, Coleman JRI, Gaspar HA, et al. Genome-wide association study identifies eight risk loci and implicates metabo-psychiatric origins for anorexia nervosa. Nat Genet. 2019;51:1207–14. 31308545 10.1038/s41588-019-0439-2 PMC6779477

[19] Gudmundsson OO, Walters GB, Ingason A, Johansson S, Zayats T, Athanasiu L, et al. Attention-deficit hyperactivity disorder shares copy number variant risk with schizophrenia and autism spectrum disorder. Transl Psychiatry. 2019;9:258. 31624239 10.1038/s41398-019-0599-y PMC6797719

[20] Verlouw JAM, Clemens E, de Vries JH, Zolk O, Verkerk AJMH, Am Zehnhoff-Dinnesen A, et al. A comparison of genotyping arrays. Eur J Hum Genet. 2021;29:1611–24. 34140649 10.1038/s41431-021-00917-7 PMC8560858

[21] Collins RL, Glessner JT, Porcu E, Lepamets M, Brandon R, Lauricella C, et al. A cross-disorder dosage sensitivity map of the human genome. Cell. 2022;185:3041–3055.e25. 35917817 10.1016/j.cell.2022.06.036 PMC9742861

[22] Bailey JA, Gu Z, Clark RA, Reinert K, Samonte RV, Schwartz S, et al. Recent segmental duplications in the human genome. Science. 2002;297:1003–7. 12169732 10.1126/science.1072047

[23] Shirley MD, Baugher JD, Stevens EL, Tang Z, Gerry N, Beiswanger CM, et al. Chromosomal variation in lymphoblastoid cell lines. Hum Mutat. 2012;33:1075–86. 22374857 10.1002/humu.22062 PMC3370055

[24] Huang AY, Yu D, Davis LK, Sul JH, Tsetsos F, Ramensky V, et al. Rare Copy Number Variants in NRXN1 and CNTN6 Increase Risk for Tourette Syndrome. Neuron. 2017;94:1101–1111.e7. 28641109 10.1016/j.neuron.2017.06.010 PMC5568251

[25] Collins RL, Brand H, Karczewski KJ, Zhao X, Alföldi J, Francioli LC, et al. A structural variation reference for medical and population genetics. Nature. 2020;581:444–51. 32461652 10.1038/s41586-020-2287-8 PMC7334194

[26] Calle Sánchez X, Helenius D, Bybjerg-Grauholm J, Pedersen C, Hougaard DM, Børglum AD, et al. Comparing Copy Number Variations in a Danish Case Cohort of Individuals With Psychiatric Disorders. JAMA Psychiatry. 2022;79:59–69. 34817560 10.1001/jamapsychiatry.2021.3392 PMC8733851

[27] Marshall CR, Howrigan DP, Merico D, Thiruvahindrapuram B, Wu W, Greer DS, et al. Contribution of copy number variants to schizophrenia from a genome-wide study of 41,321 subjects. Nat Genet. 2017;49:27–35. 27869829 10.1038/ng.3725 PMC5737772

[28] Lek M, Karczewski KJ, Minikel EV, Samocha KE, Banks E, Fennell T, et al. Analysis of protein-coding genetic variation in 60,706 humans. Nature. 2016;536:285–91. 27535533 10.1038/nature19057 PMC5018207

[29] Kendall KM, Rees E, Bracher-Smith M, Legge S, Riglin L, Zammit S, et al. Association of Rare Copy Number Variants With Risk of Depression. JAMA Psychiatry. 2019;76:818–25. 30994872 10.1001/jamapsychiatry.2019.0566 PMC6583866

[30] Fu JM, Satterstrom FK, Peng M, Brand H, Collins RL, Dong S, et al. Rare coding variation provides insight into the genetic architecture and phenotypic context of autism. Nat Genet. 2022;54:1320–31. 35982160 10.1038/s41588-022-01104-0 PMC9653013

[31] Bryois J, Skene NG, Hansen TF, Kogelman LJA, Watson HJ, Liu Z, et al. Genetic identification of cell types underlying brain complex traits yields insights into the etiology of Parkinson’s disease. Nat Genet. 2020;52:482–93. 32341526 10.1038/s41588-020-0610-9 PMC7930801

[32] Kaplanis J, Samocha KE, Wiel L, Zhang Z, Arvai KJ, Eberhardt RY, et al. Evidence for 28 genetic disorders discovered by combining healthcare and research data. Nature. 2020;586:757–62. 33057194 10.1038/s41586-020-2832-5 PMC7116826

[33] Singh T, Poterba T, Curtis D, Akil H, Al Eissa M, Barchas JD, et al. Rare coding variants in ten genes confer substantial risk for schizophrenia. Nature. 2022;604:509–16. 35396579 10.1038/s41586-022-04556-w PMC9805802

[34] Bergen, Ploner SE, Howrigan A, CNV Analysis Group and the Schizophrenia Working Group of the Psychiatric Genomics Consortium D, O’Donovan MC, Smoller JW, et al. Joint Contributions of Rare Copy Number Variants and Common SNPs to Risk for Schizophrenia. Am J Psychiatry. 2019;176:29–35. 30392412 10.1176/appi.ajp.2018.17040467 PMC6408268

[35] Cross-Disorder Group. of the Psychiatric Genomics Consortium. Electronic address: plee0@mgh.harvard.edu, Cross-Disorder Group of the Psychiatric Genomics Consortium. Genomic Relationships, Novel Loci, and Pleiotropic Mechanisms across Eight Psychiatric Disorders. Cell. 2019;179:1469–1482.e11. 31835028 10.1016/j.cell.2019.11.020 PMC7077032

[36] Sirugo G, Williams SM, Tishkoff SA. The Missing Diversity in Human Genetic Studies. Cell. 2019;177:26–31. 30901543 10.1016/j.cell.2019.02.048 PMC7380073

[37] Liu D, Meyer D, Fennessy B, Feng C, Cheng E, Johnson JS, et al. Schizophrenia risk conferred by rare protein-truncating variants is conserved across diverse human populations. Nat Genet. 2023;55:369–76. 36914870 10.1038/s41588-023-01305-1 PMC10011128

[38] Babadi M, Fu JM, Lee SK, Smirnov AN, Gauthier LD, Walker M, et al. GATK-gCNV enables the discovery of rare copy number variants from exome sequencing data. Nat Genet. 2023;55:1589–97. 37604963 10.1038/s41588-023-01449-0 PMC10904014

