# Supplementary material for: A burden of rare copy number variants in obsessive-compulsive disorder
Source: Mol Psychiatry. 2024 Oct 27;30(4):1510–7. doi: 10.1038/s41380-024-02763-7 (PMC11919692; doi:10.1038/s41380-024-02763-7)
Supplement: Supplementary file 1 — Supplementary Information [file 41380_2024_2763_MOESM1_ESM.docx]

Table of Contents

[**Figure S1 : Principal Component Analysis of common variation in NORDiC cases and unaffected controls. 2**](#_heading=)

[**Figure S2 : Principal Component Analysis of common variation in NORDiC Norwegian and Swedish samples. 3**](#_heading=)

[**Figure S3 : Principal Components 9-20 from Norwegian and Swedish samples. 4**](#_heading=)

[**Figure S4 : Scree plot for Principal Component Analysis of common variation. 5**](#_heading=)

[**Figure S5 : Log R ratio standard deviation mean and standard deviation per dataset. 6**](#_heading=)

[**Figure S6 : Mean and standard deviation for raw CNV call count per dataset. 7**](#_heading=)

[**Figure S7 : Mean and standard deviation for analysis-ready CNV call count per dataset. 8**](#_heading=)

[**Figure S8 : QQ plots for locus-based association tests using CNVs 30-100kb in size. 9**](#_heading=)

[**Figure S9 : QQ plots for locus-based association tests using CNVs between 100kb and 500kb in size. 10**](#_heading=)

[**Figure S10 : Leave-one-out analyses of global CNV burden in cases versus controls. 11**](#_heading=)

[**Figure S11 : Covariate leave-one-out analyses of global CNV burden in cases versus controls. 12**](#_heading=)

[**Figure S12 : Global CNV burden in cases versus controls stratified by CNV size. 13**](#_heading=)

[**Figure S13 : Global CNV burden in cases versus controls stratified by CNV frequency. 14**](#_heading=)

[**Figure S14 : Burden of CNVs in overlapping genes that are haplosensitive or triplosensitive. 15**](#_heading=)

[**Figure S15 : ​​Global genic CNV burden in Norwegian versus Swedish samples. 16**](#_heading=h.67pcqa3uhz7v)

[**Figure S16 : Global genic CNV burden in male versus female samples. 17**](#_heading=h.uq464pwtbeei)

[**Figure S17 : Global burden of noncoding CNV bases in cases versus controls, stratified by mammalian constraint. 18**](#_heading=)

# Figure S1 : Principal Component Analysis of common variation in NORDiC cases and unaffected controls.


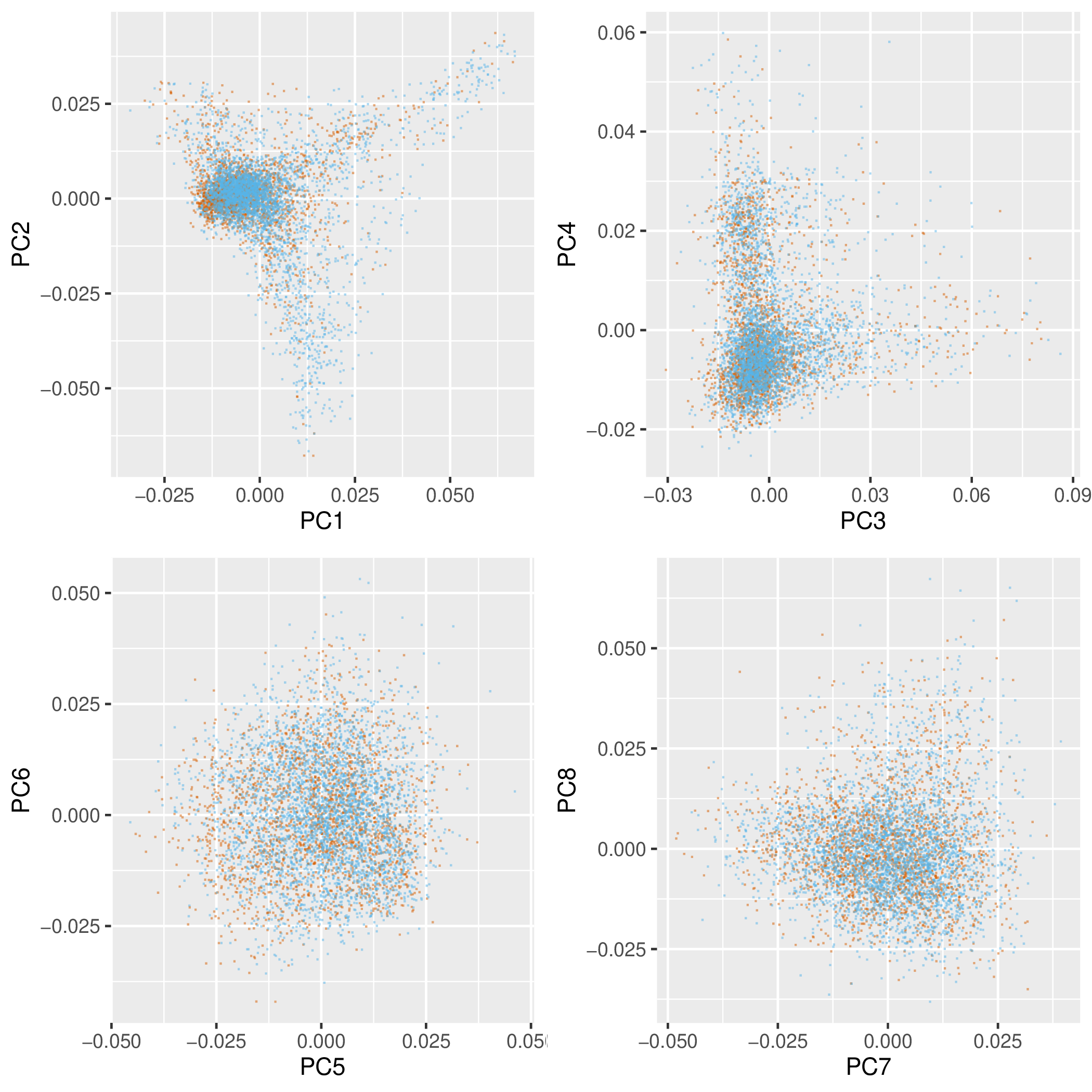


Figure 1 shows a Principal Component Analysis of common genetic variation in NORDiC cases (orange) and unaffected controls (blue). There is no visible stratification of cases and controls along the main principal components.

# Figure S2 : Principal Component Analysis of common variation in NORDiC Norwegian and Swedish samples.


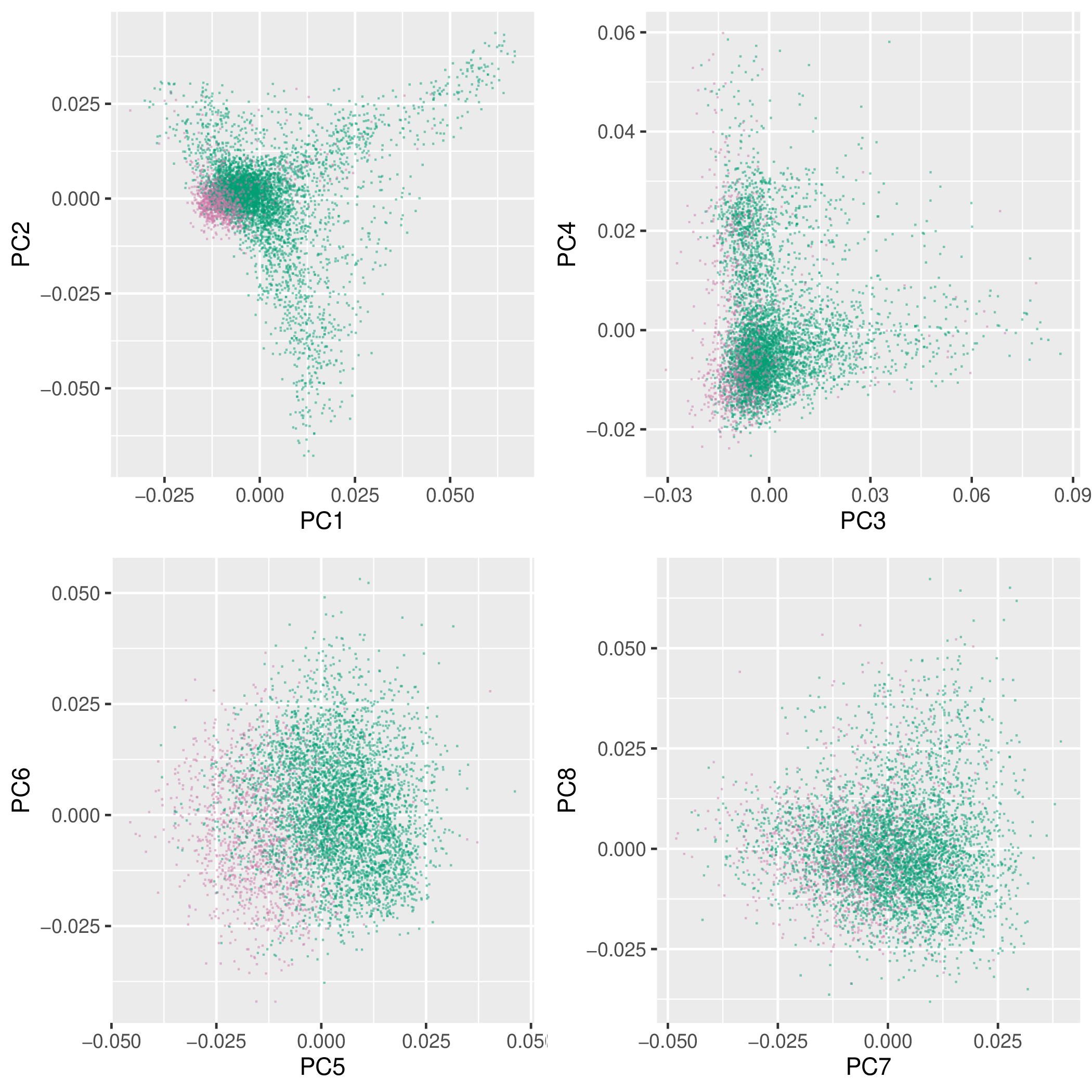


Figure 2 shows results from the same Principal Component Analysis as that depicted in Figure S1, but here, samples are colored by country of origin. Swedish samples are in green, and Norwegian samples are in purple. Here, we can see that Swedish and Norwegian samples do separate along some of the main principal components (PCs 1 and 5 in particular). There is Swedish and Norwegian representation in both the case and control groups, but these results make it clear that controlling for Swedish vs. Norwegian ancestry is appropriate in this analysis, be it via use of appropriate PCs as covariates or via meta-analysis of Sweden-only and Norway-only summary statistics.

# Figure S3 : Principal Components 9-20 from Norwegian and Swedish samples.


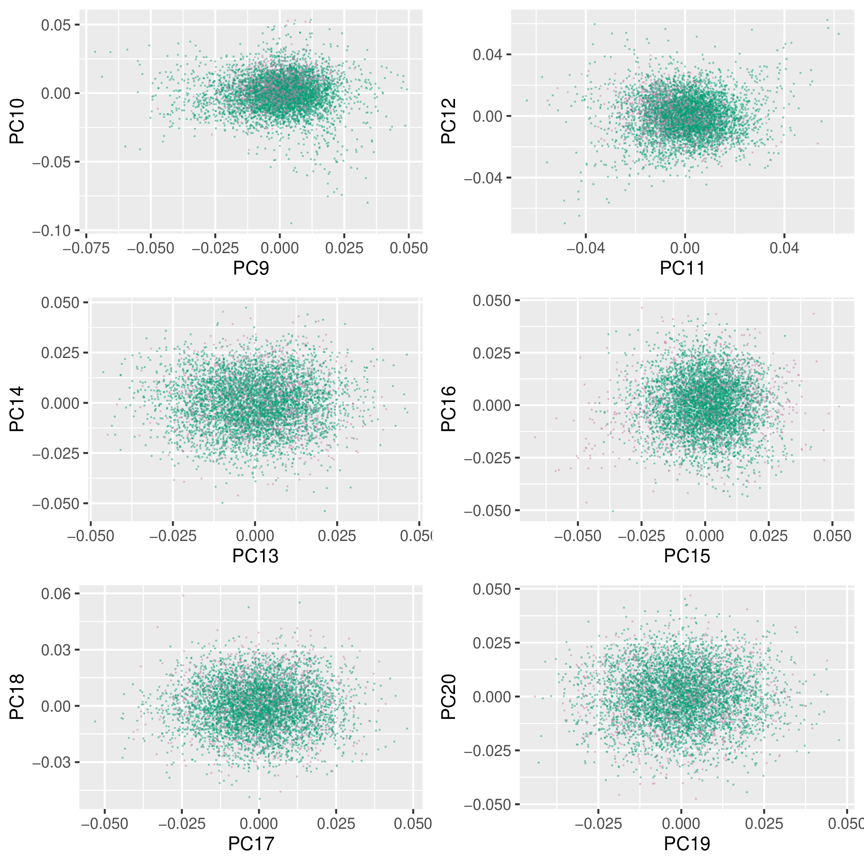


Figure 3 depicts the results from Principal Components 9-20 for the same Principal Component Analysis as that in Figure S1 and S2, where only PCs 1-8 are shown. As in Figure S2, Swedish samples are in green, and Norwegian samples are in purple. We do not observe total separation of Swedish and Norwegian samples across any of these components shown.

# Figure S4 : Scree plot for Principal Component Analysis of common variation.


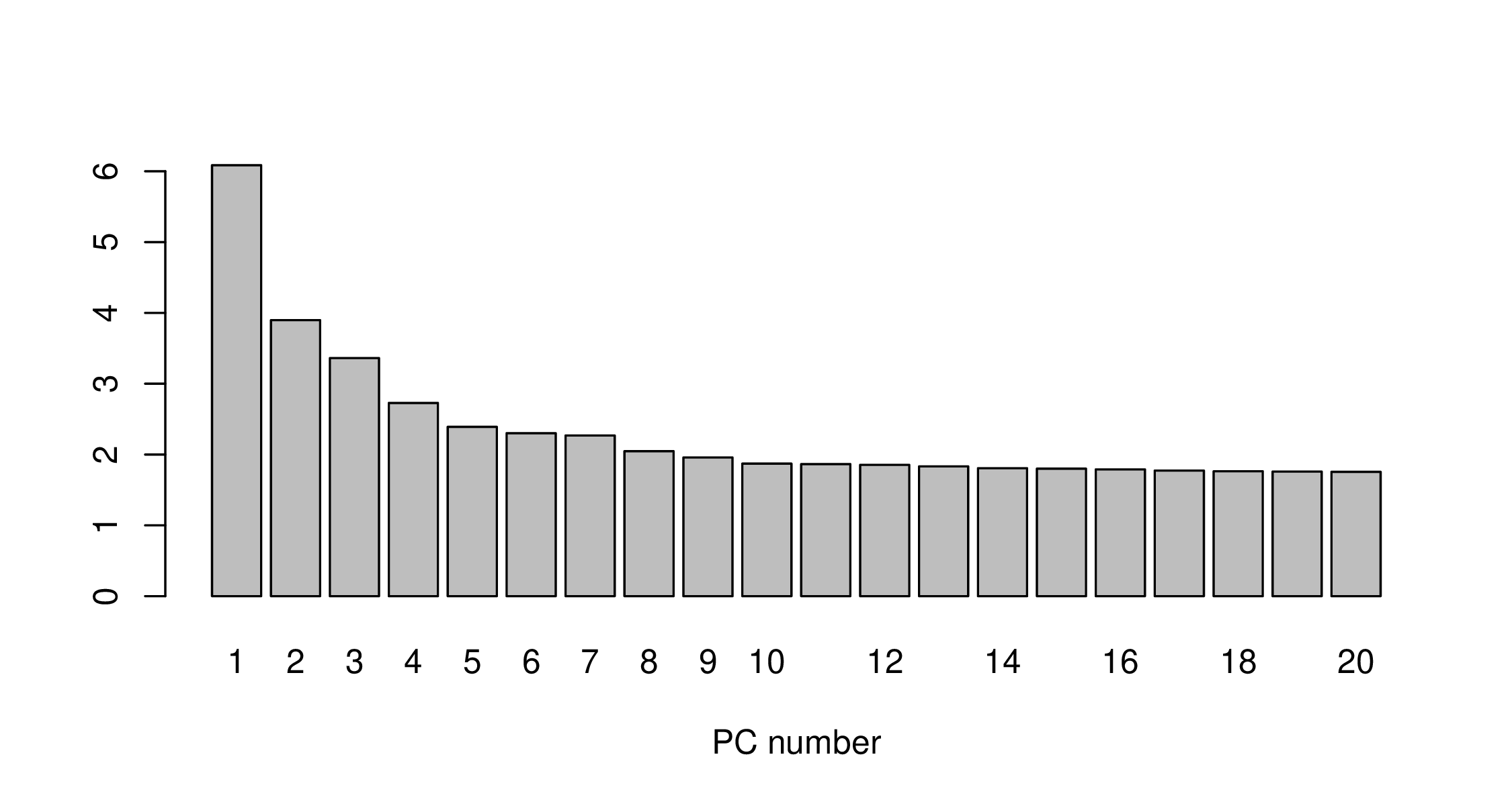


Figure 4 depicts a Scree plot for the Principal Component Analysis of common variation, showing the amount of variance explained by each of the principal components computed. We see that the large majority of the variance explained in the data comes specifically from the first 5 PCs. We use these PCs as covariates in our regression models by default, along with the subset of PCs 6-20 that showed nominal association with both case status and raw CNV count (P<0.01 for both).

# Figure S5 : Log R ratio standard deviation mean and standard deviation per dataset.


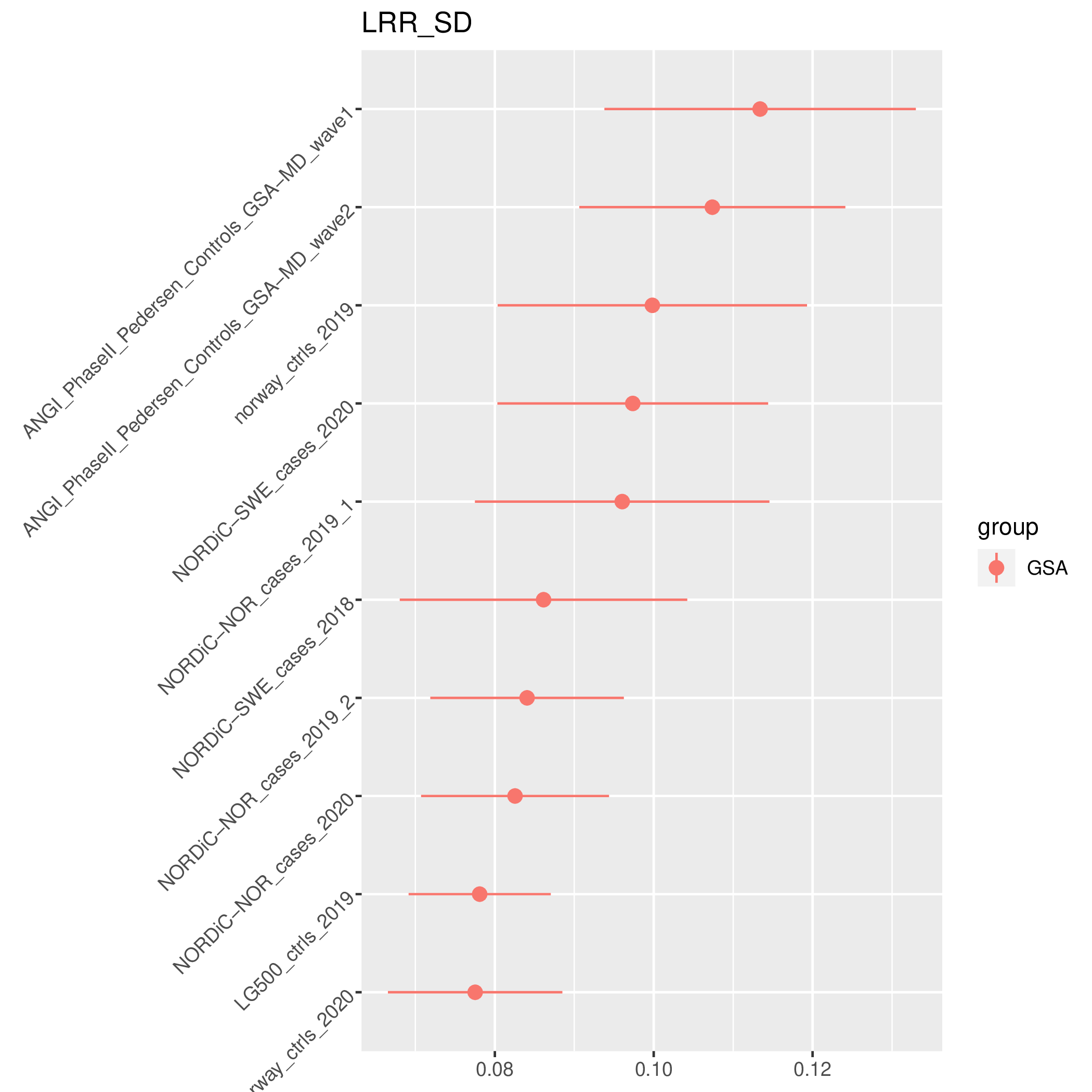


Figure 5 shows the mean and standard deviation of the Log R Ratio Standard Deviation (LRRSD) metrics per input dataset. In general, LRRSD metrics are well controlled, with no individual dataset having a mean LRRSD greater than 0.12. It is notable that the both ANGI datasets appear to have slightly higher LRRSD values than the other datasets, particularly since for these datasets, we received them in the form of pre-clustered VCFs with intensity metrics, and were unable to put them down precisely the same gtc2vcf pipeline as other cohorts.

# Figure S6 : Mean and standard deviation for raw CNV call count per dataset.


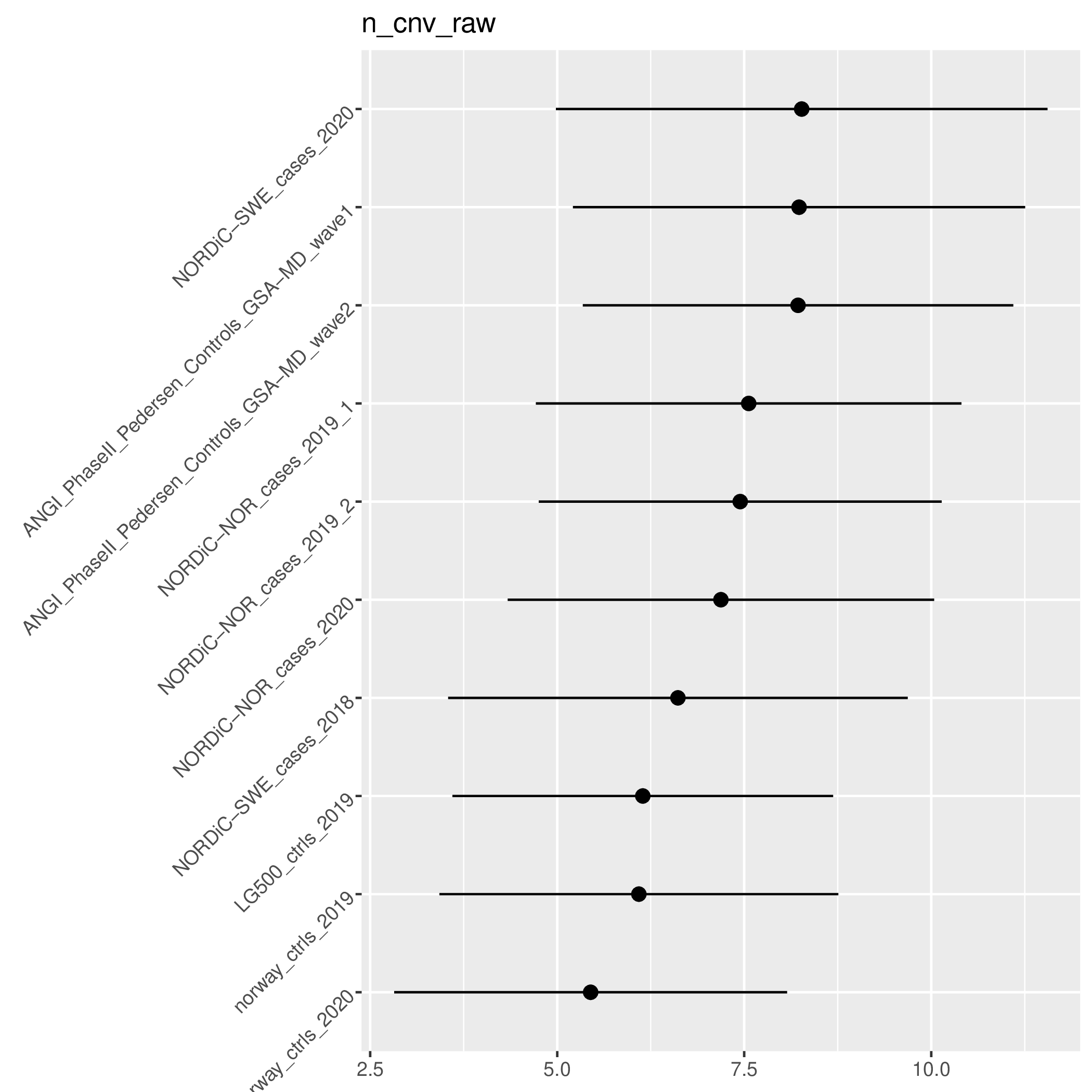


Figure 6 shows the mean and standard deviation of raw CNV call count per input dataset used in this study. There is notable variance in raw counts across datasets, necessitating stringent quality control of CNV calls included in comparisons.

# Figure S7 : Mean and standard deviation for analysis-ready CNV call count per dataset.


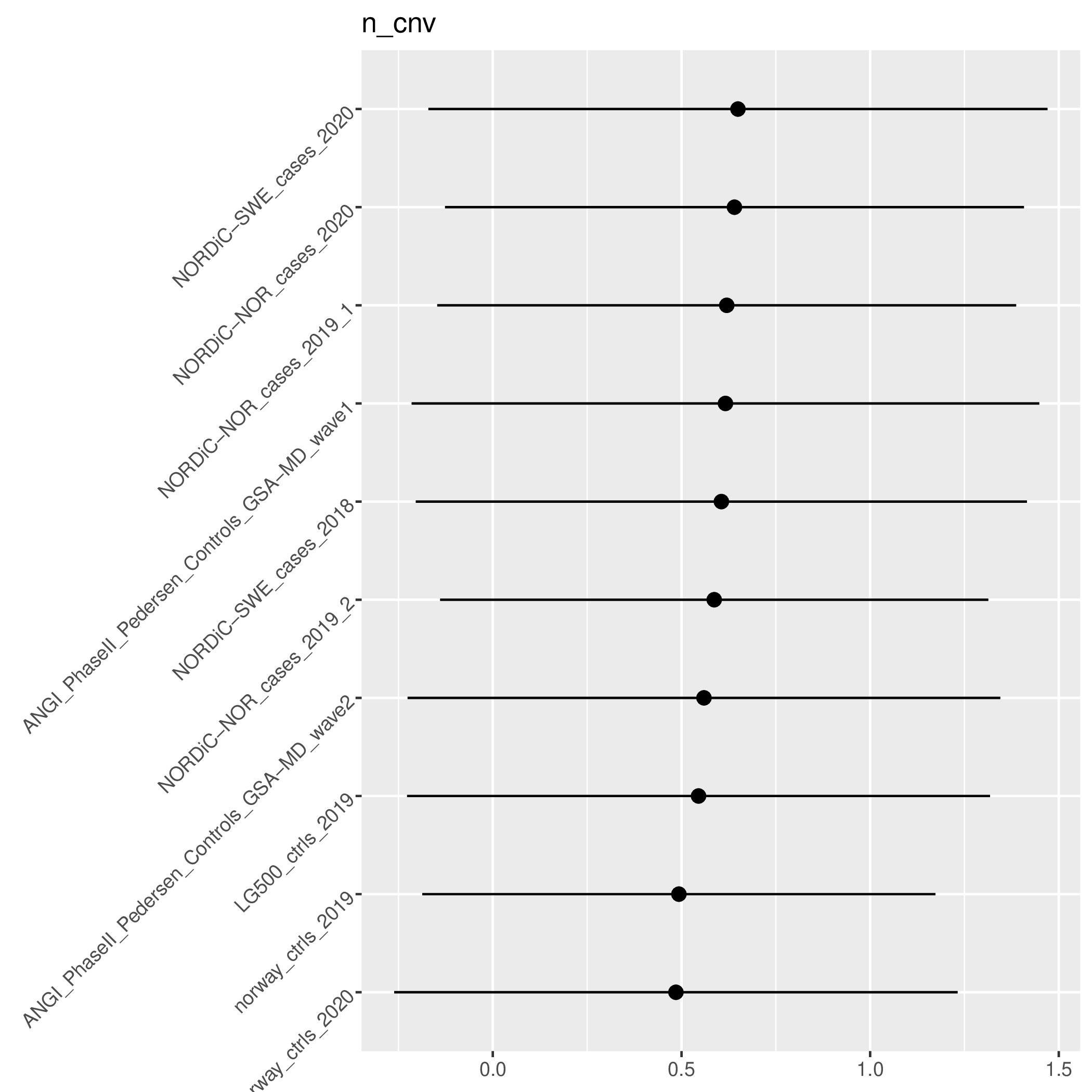


Figure 7 shows the mean and standard deviation of post-QC, analysis-ready CNV call counts per dataset. We see that here, relative to raw pre-QC CNV counts shown in Figure S6, call count metrics across separate datasets are far more comparable, with mean call counts per dataset ranging from around 0.5 to 0.65.

# Figure S8 : QQ plots for locus-based association tests using CNVs 30-100kb in size.


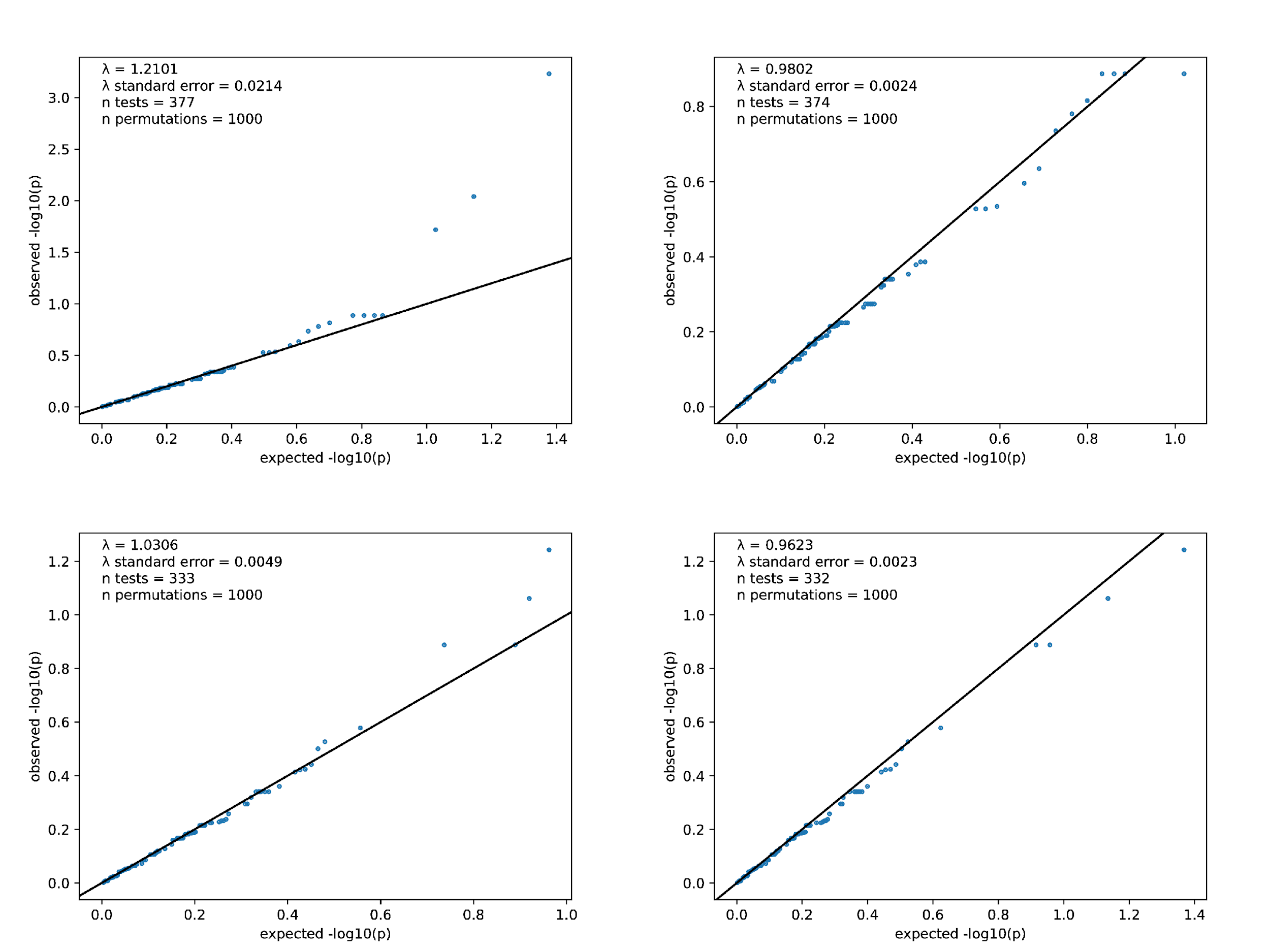


Figure 8 shows QQ plots for locus-based association tests using CNVs between 30kb and 100kb in size, with the intended purpose of finding loci that harbor clusters of case or control-biased CNV calls that are more likely from batch effects than true biology. We find evidence of some p-value inflation when producing a QQ plot specific to deletions in this size bin (top left). After exclusion of CNVs in this size bin that overlap these 3 specific loci, the inflation for deletions is gone (top right). There is not the same amount of obvious p-value inflation for duplications (bottom left), though we see that the genomic inflation estimate lambda for duplications goes down after duplications from these 3 prior mentioned loci are excluded (bottom right).

# Figure S9 : QQ plots for locus-based association tests using CNVs between 100kb and 500kb in size.


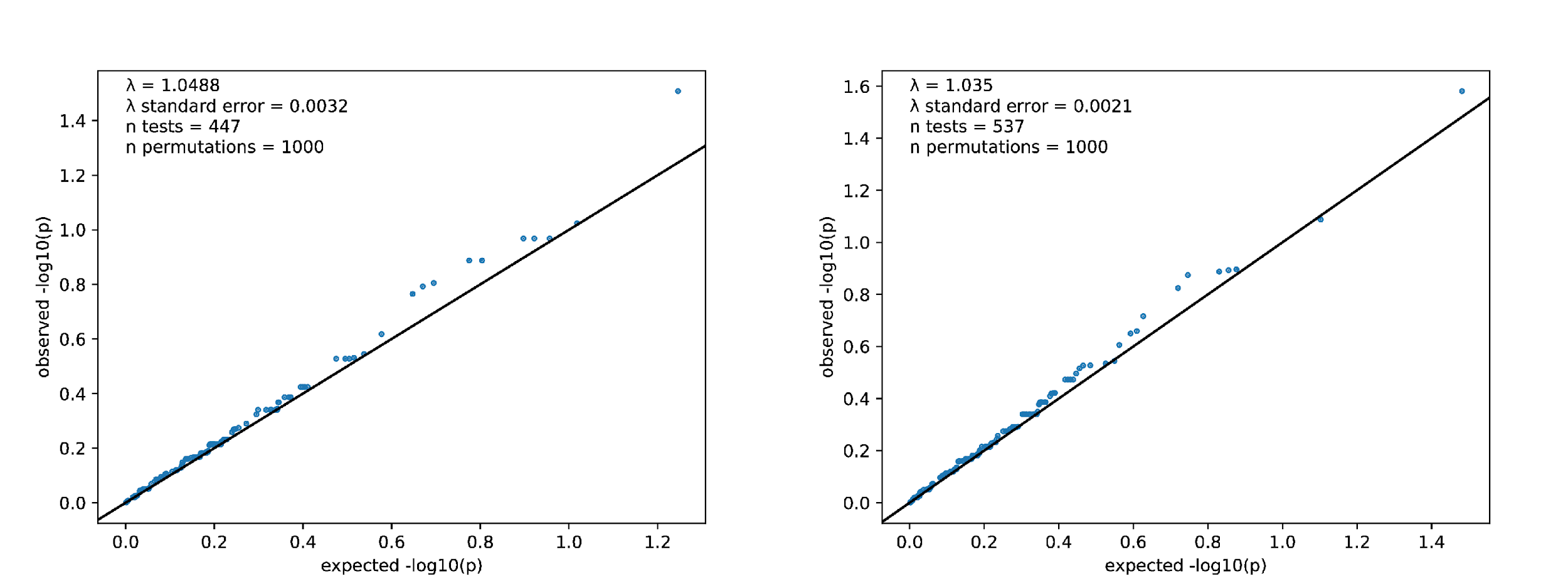


Figure 9 shows a QQ plot of locus-based association tests for deletions (left) and duplications (right) specifically for CNVs between 100 and 500 kb in size. There is no evidence of unreasonable p-value inflation across either set of tests.

# Figure S10 : Leave-one-out analyses of global CNV burden in cases versus controls.


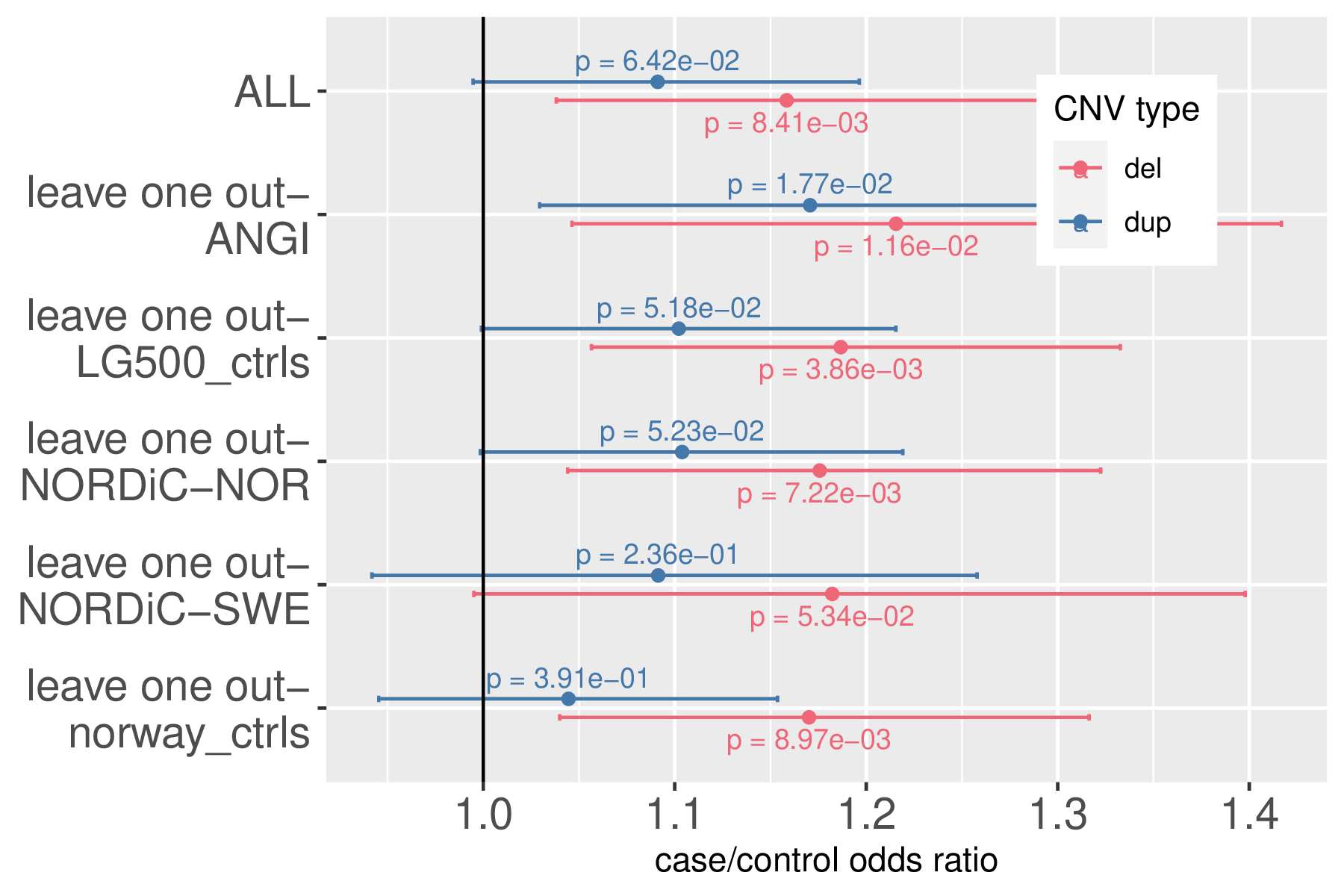


Figure 10 depicts the odds ratio estimate (dots) and 95% confidence interval (bars) for association between case status and each additional analysis-qualifying CNV call, in both the global case/control comparison and in leave-one-out analyses of specific cohorts included. While the case duplication excess is unstable across leave-one-out analyses, the case deletion excess appears to be stable (P<0.05 for all control leave-one-out comparisons, and P<0.1 for all case leave-one-out comparisons).

# Figure S11 : Covariate leave-one-out analyses of global CNV burden in cases versus controls.


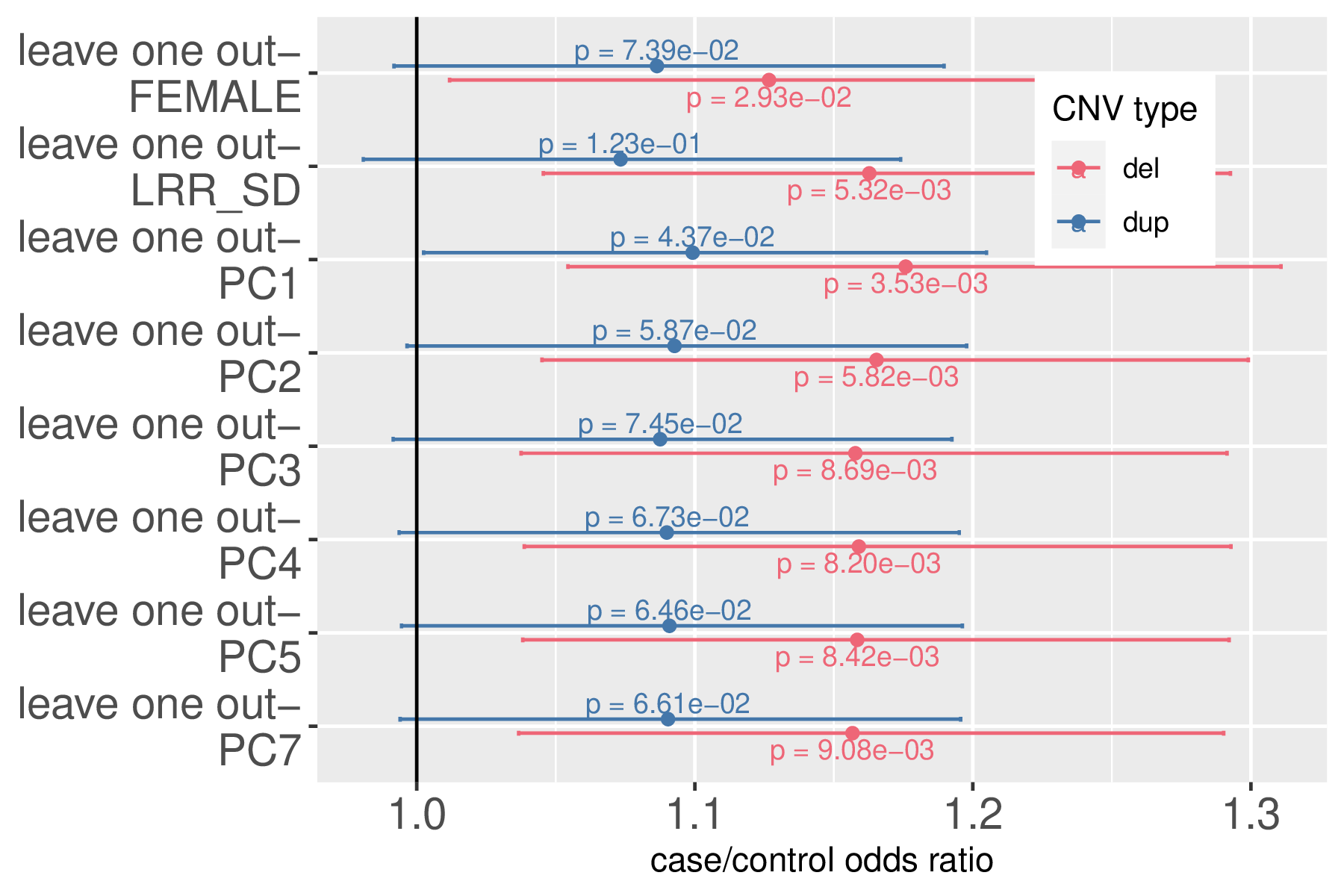


Figure 11 shows the results of leave-one-out analyses of global CNV burden versus case status, specific to individual covariates used in the regression model. Results are depicted in the form of odds ratio estimates (dots) and 95% confidence intervals for the estimates (bars). Sex appears to be the covariate that has the greatest impact on association strength, likely due to the fact that the ANGI dataset is heavily female skewed (97.9% of cohort).

# Figure S12 : Global CNV burden in cases versus controls stratified by CNV size.


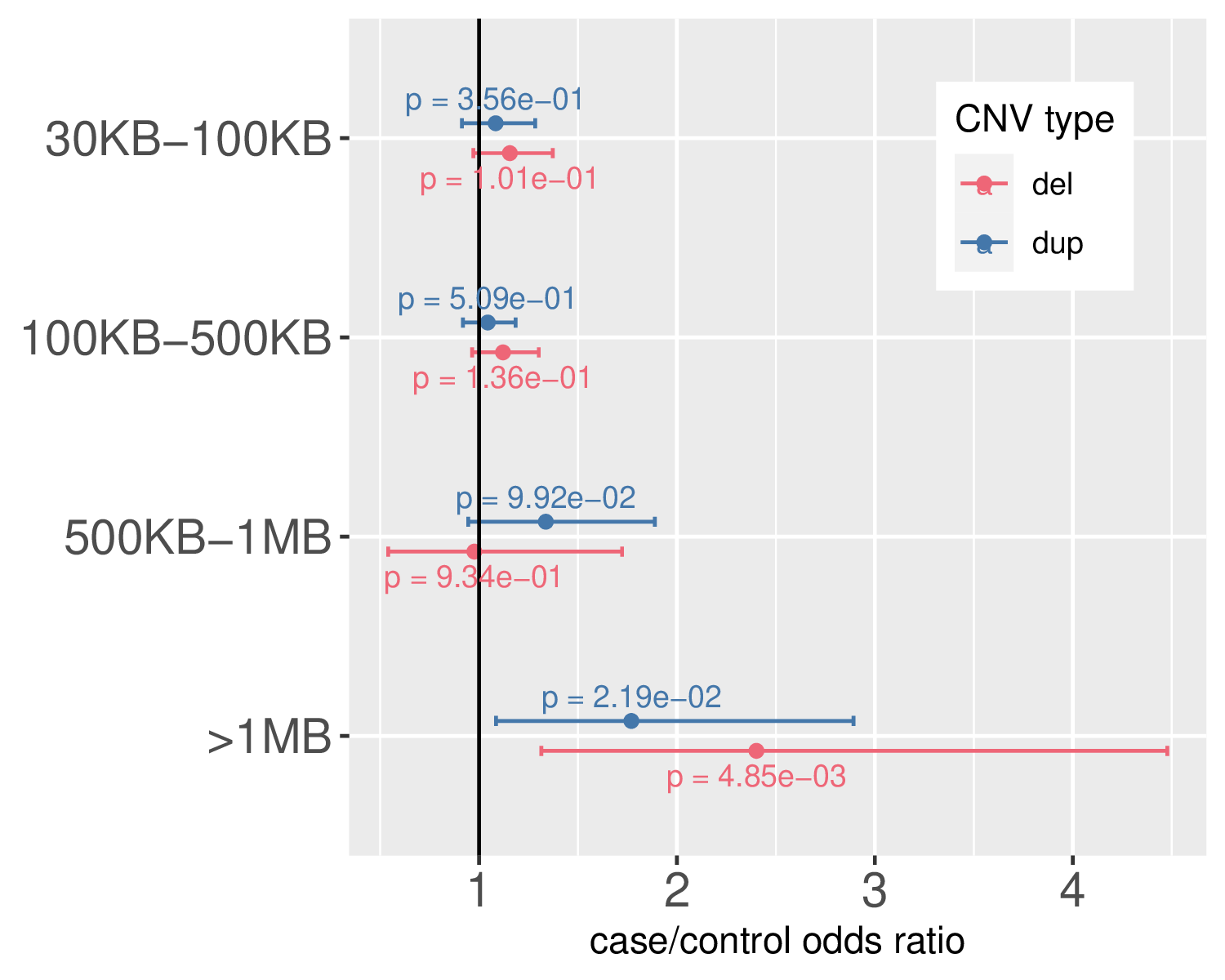


Figure 12 depicts association test results between CNV deletion (red) and duplication (blue) burden and OCD case status, in the form of odds ratio estimates (dots) and 95% confidence intervals (bars). As has been seen in similar studies of other psychiatric case cohorts, the CNVs that confer the largest amount of case relative risk are those that are large (>1MB in size).

# Figure S13 : Global CNV burden in cases versus controls stratified by CNV frequency.


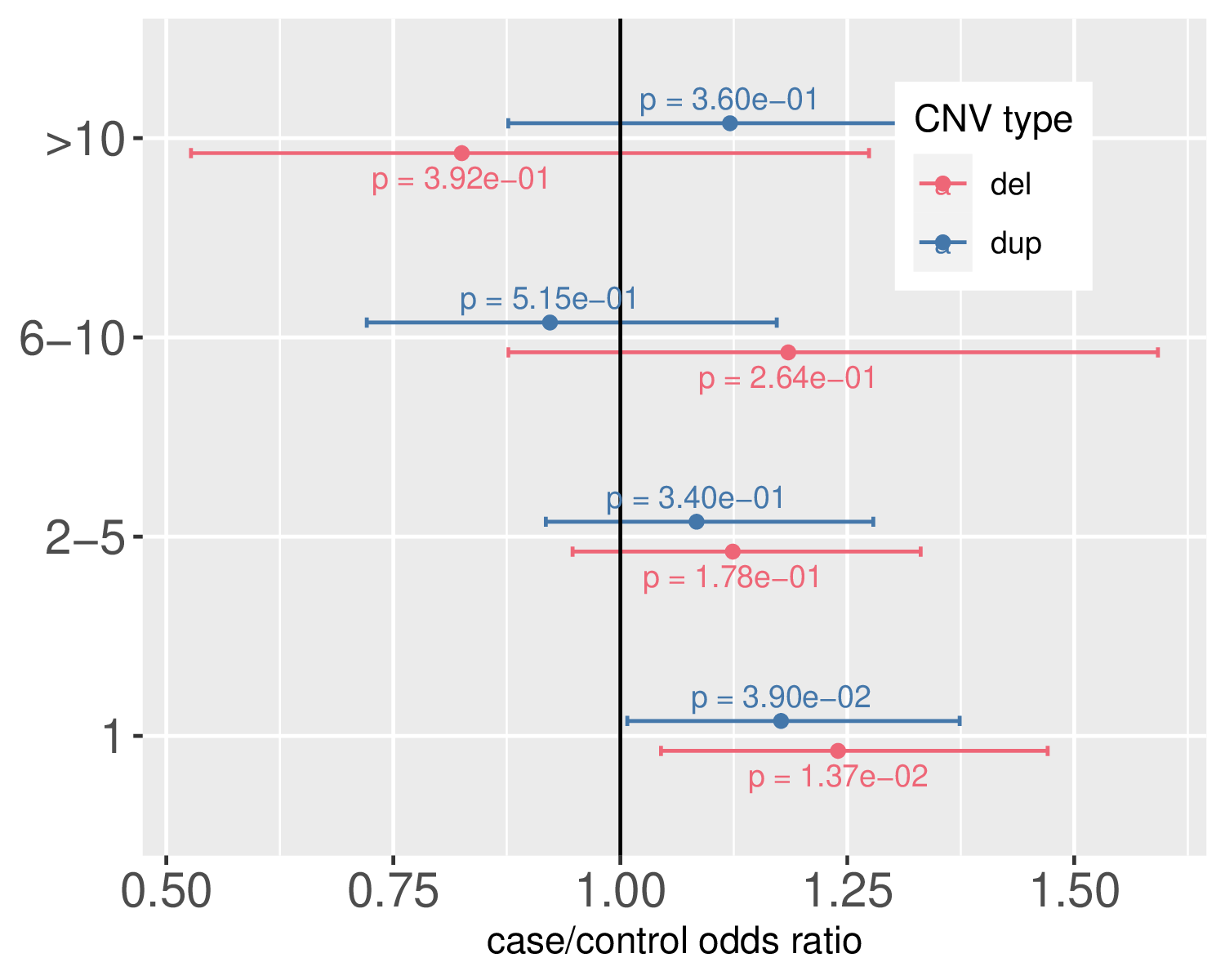


Figure 13 shows association test results between CNV count and case status, stratified by CNV frequency. As before, odds ratio estimates are depicted with dots, and 95% confidence intervals are depicted with bars. We see that association test statistics for both deletions (red) and duplications (blue) are strongest for singleton CNVs, which are only observed once in the case/control cohort.

# Figure S14 : Burden of CNVs in overlapping genes that are haplosensitive or triplosensitive.


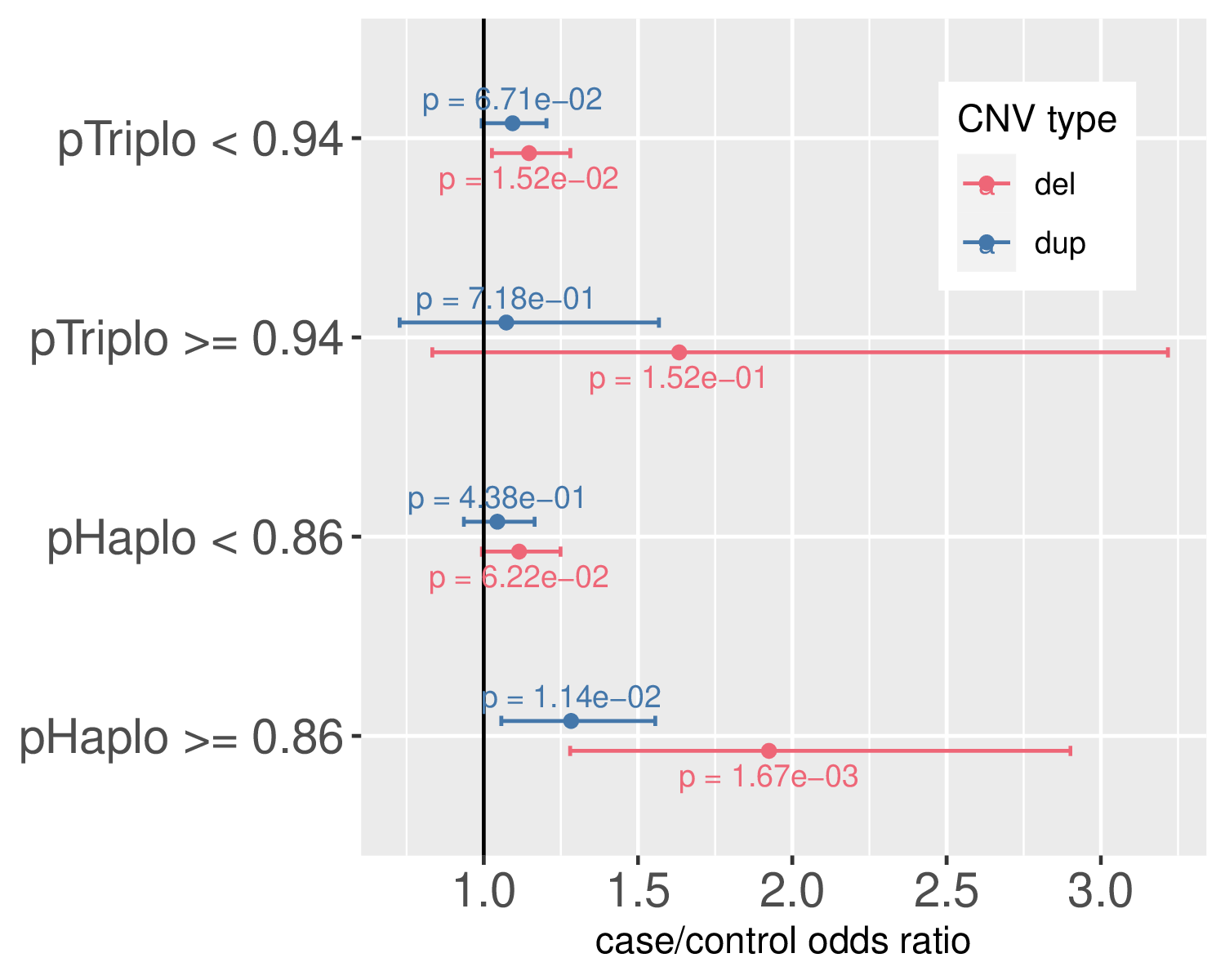


Figure 14 shows the results of association tests between CNV count and case status, stratified by whether or not a gene is impacted that is triplosensitive or haplosensitive based on a recently described CNV dosage sensitivity metric. As before, dots are the odds ratio estimates, and bars are the 95% confidence intervals for the estimates. The triplosensitivity metric does not prove effective in identifying genes enriched for CNV burden in OCD cases, whereas the haplosensitivity metric does appear effective in doing this for both duplications and deletions.

# Figure S15 : ​​Global genic CNV burden in Norwegian versus Swedish samples.


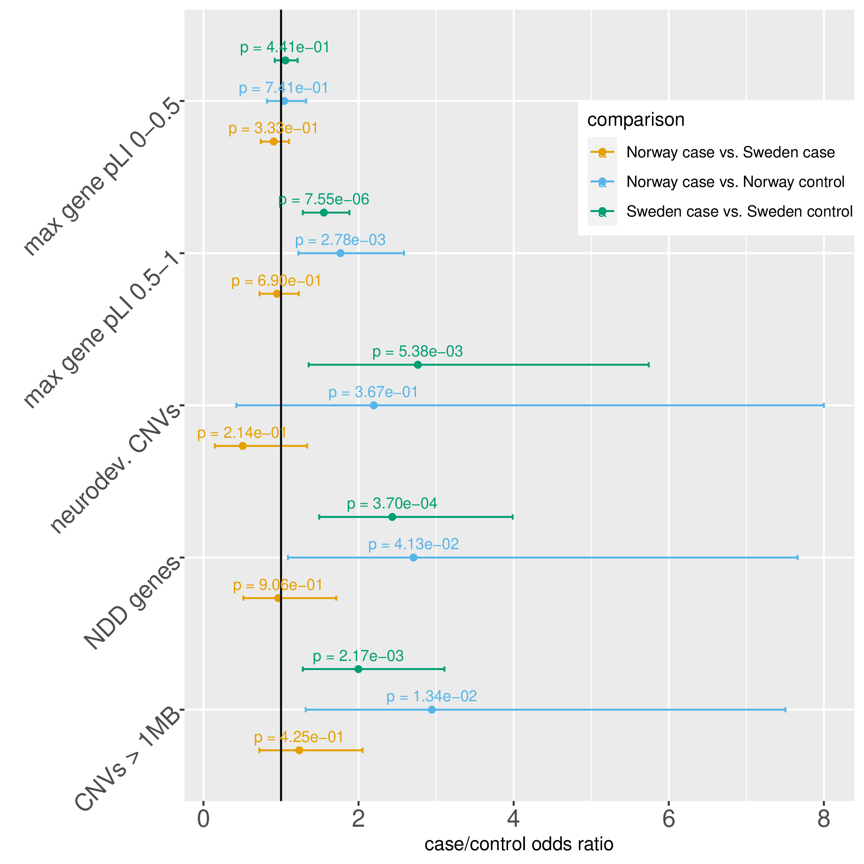


Figure 15 depicts the results of association tests between various country-of-origin-based outcomes (Sweden/Norway, see legend above) and total count of CNVs for various categories of genic CNV burden, with Log-R Ratio Standard Deviation and nongenic CNV count as covariates. Odds ratio estimates are depicted (dots) along with 95% confidence intervals (bars). We fail to detect any CNV burden difference for any outcome considered for CNVs only impacting non-constrained genes (max pLI<0.5). In general, for Sweden-only and Norway-only case/control comparisons, we detect consistent OCD case burden relative to controls for all other categories tested. We fail to detect a significant difference in burden in Norwegian versus Swedish cases across these categories containing constrained genes.

# Figure S16 : Global genic CNV burden in male versus female samples.

**
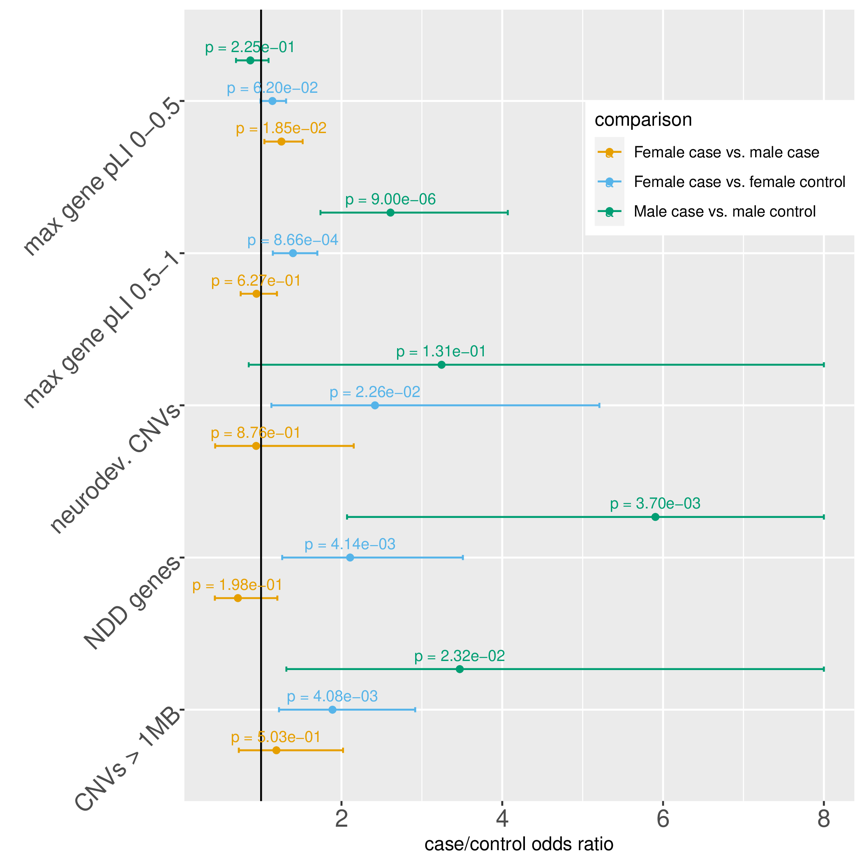
**

Figure 16 depicts the results of association tests between various sex-based outcomes (see legend above) and total count of CNVs for various categories of genic CNV burden, with Log-R Ratio Standard Deviation and nongenic CNV count as covariates. Odds ratio estimates are depicted (dots) along with 95% confidence intervals (bars). While we see comparable burden across all outcomes of genic CNVs with max probability of loss of function intolerance (pLI) < 0.5 (ie. non-constrained), all other categories indicate 1) sex-stratified case CNV excess relative to sex matched controls, and 2) a lack of detectable difference between male and female cases. While there was no detectable difference of CNV burden between male and female cases across constrained categories, we do note interestingly that the effect size estimates appear larger in general for male cases than for female cases.

# Figure S17 : Global burden of noncoding CNV bases in cases versus controls, stratified by mammalian constraint.


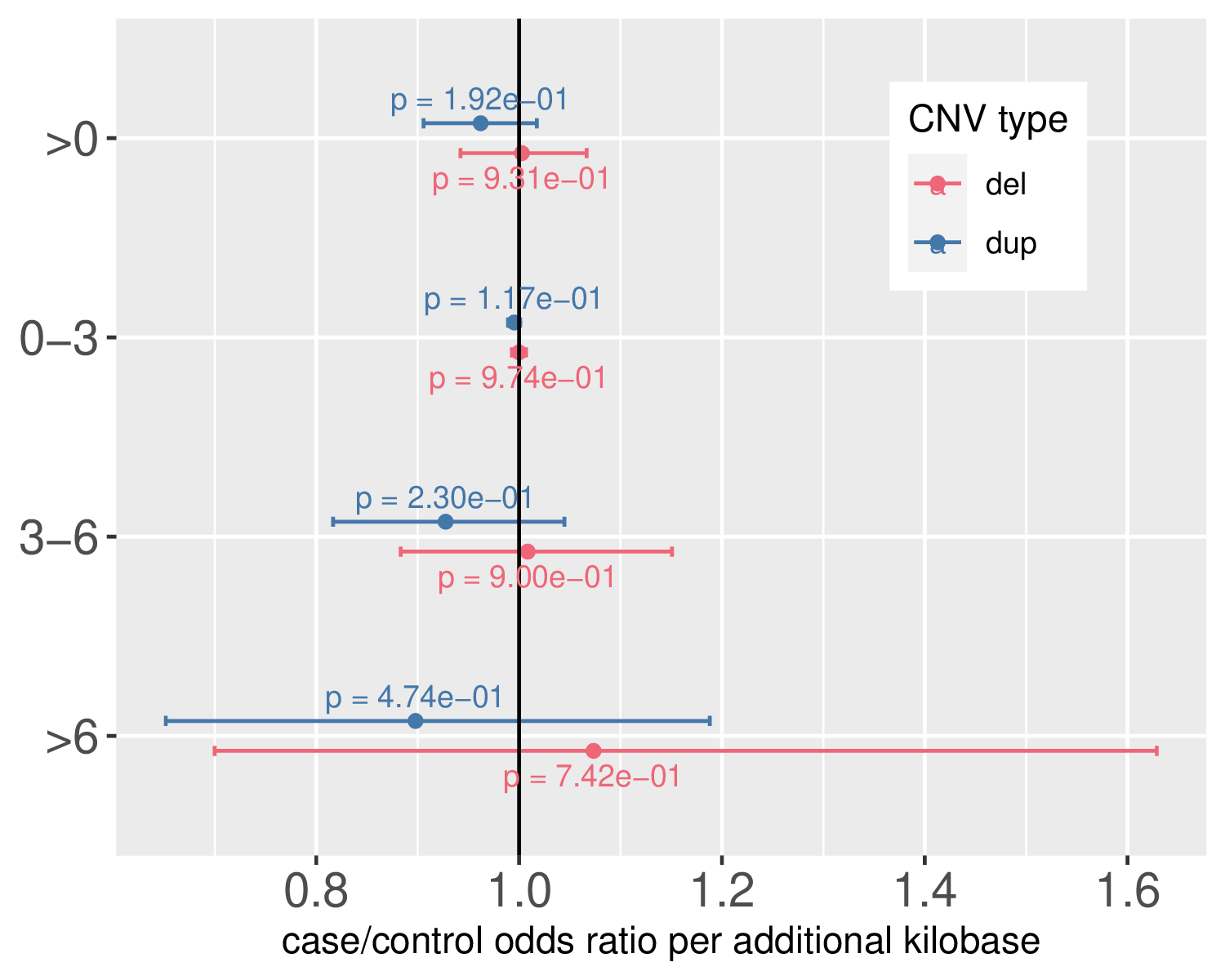


Figure 17 depicts association statistics between cumulative number of noncoding bases overlapping a CNV call and case status, with base counts stratified by mammalian constraint score bin. Odds ratio estimates are depicted (dots) along with 95% confidence intervals (bars). There is no significant association between burden and case status across any of these constraint bins.
