# Supplementary material for: A burden of rare copy number variants in obsessive-compulsive disorder
Source: Mol Psychiatry. 2024 Oct 27;30(4):1510–7. doi: 10.1038/s41380-024-02763-7 (PMC11919692; doi:10.1038/s41380-024-02763-7)
Supplement: Supplementary file 2 — Supplemental Methods [file 41380_2024_2763_MOESM2_ESM.pdf]

SUPPLEMENTAL METHODS

Conversion of intensity data to VCFs using the gtc2vcf pipeline 1
Sample-level quality control procedure 2
CNV calling procedure 3
B allele frequency validation procedure 4
Tests for loci harboring CNV calls that drive genomic inflation 5
Power analysis 6
Association tests between CNV burden, country-of-origin and sex 8
Pathway enrichment analysis 9
References 10

Conversion of intensity data to VCFs using the gtc2vcf pipeline

We used the gtc2vcf pipeline (https://github.com/freeseek/gtc2vcf) to convert intensity data files into VCFs that contained hard-call genotypes and intensity information per sample-variant combination. For each dataset, the appropriate canonical BPM file and EGT cluster file specific to the input GSA version (v1, v2, v3, DeCODE) were obtained and used as input alongside the IDATs. In brief, the 'gencall' command in the Illumina command line utility (iaap-cli, v1.1.0-1.1.0-sha.80d7e5b3d9c1fdfc2e99b472a90652fd3848bbc7) was used to produce GTC files for each dataset processed from input IDATs, BPM and EGT files, and the 'gtc2vcf' plugin for bcftools (version 2020-01-14 from https://github.com/freeseek/gtc2vcf) was used with the '—adjust-clusters' option turned to adjust cluster centers in a manner that is specific to each input dataset and produce BCF files with genotype calls and intensity values per dataset. We did not have raw data for ANGI controls, and thus were unable to put them through this exact same pipeline. We did have VCF files containing calls and intensity data for these samples, and were thus well-positioned to extract data needed for case/control study.

We next formed a single merged dataset consisting of markers found across all input data using PLINK v1.90b4.9. Before performing this merger, we aligned markers across datasets by their

combination of chromosome, position, SNP ID, reference allele and alternate allele. This process was made easier by the fact that all data were processed relative to the same reference FASTA file (GRCh37 human reference build), and so for a given SNP marker, reference and alternate alleles match across datasets. The Norwegian DeCODE control datasets were an exception to this, and we had to liftover their marker coordinates GRCh38 to GRCH37 using the Picard v2.25.2 command 'LiftoverVcf'.

Sample-level quality control procedure

Sample biological QC was performed on the merged genotype data, starting with checks on missingness, sex and relatedness using PLINK v1.90b4.9. We first removed a total of 64 samples that had a genotype missingness rate in the merged dataset > 0.02. We next performed concordance checks between reported and data-derived sex. We removed 25 cases where either the data-derived sex was undefined, or was defined but did not match a clearly defined reported sex for that sample. From controls, we removed 7 samples where an explicit sex could not be clearly derived from array data. Next, we computed pairwise relatedness using PLINK's '—genome' function with 239,709 LD-pruned autosomal SNPs. We performed relatedness pruning, controlling for instances of 1) identified samples with a mean pihat > 0.1 with other samples, 2) sample pairs that are duplicates based on pihat > 0.95, and 3) sample pairs that are relatives based on pihat > 0.2. After these pruning steps we were left with 2624 cases and 4096 controls.

We next performed ancestry-based QC on the samples using 1000 genomes phase 3 genotypes (minor allele frequency > 0.05) as training data. We identified a total of 210,149 autosomal variants in 1000 genomes phase 3 that had a minor allele frequency > 0.05 and were also found in our merged case/control data, and merged the samples together on these variants. We then performed principal component analysis (PCA) on LD-pruned genetic data and extracted eigenvectors across the first 20 PCs. We used the 1000 genomes phase 3 samples with ancestry classifications as training data for a random forest model computing the probability of a given

sample belonging to each of the 5 ancestries. We subsetted on samples that had a probability of belonging to the EUR (European) ancestry group > 0.95. We then performed PCA on EUR-labeled case/control data, and conducted a round of outlier pruning where we removed samples > 6 standard deviations (SD) from the mean across any of the first 20 PCs. We were left with 2339 cases and 3827 controls after pruning.

We next performed sample pruning based on observed versus expected heterozygosity rates per sample, calculated using PLINK v1.90b4.9. We removed samples where the heterozygosity F statistic was over 3 SD from the mean computed across all samples. This final QC step left us with a total of 2325 cases and 3790 controls suitable for inclusion in a comparison of CNV burden between OCD cases and controls.

CNV calling procedure

We called CNVs on all NORDiC OCD cases and Swedish and Norwegian controls which we had sample-level Log R ratio (LRR) and B allele frequency (BAF) data for. We focused CNV calling on a set of 537,278 variants common to all input data derived from the genotype merger process described previously. For each input sample, we produced a file containing each SNP marker, its GRCH37 coordinates and the corresponding BAF and LRR value for that marker in that sample.

We first produced a sample-level CNV callset using PennCNV v1.0.5. We used the PennCNV command 'cal_gc_snp.pl' to produce a required gcmodel file using the set of 537,278 input variants and a PennCNV-provided file 'hg19.gc5Base_sorted.txt'. We next controlled for sample-level waviness of intensity values using the PennCNV command 'genomic_wave.pl', and the gcmodel file as input. Wave-adjusted intensity files were then used as input for the PennCNV command 'compile_pfb.pl' to produce population of B allele (PFB) files per dataset, to adjust for the BAF for each marker inherent to each input dataset. Each genomic wave-adjusted sample-

level file which listed BAF and LRR per marker was used as input for the PennCNV command 'detect_cnv.pl', along with the described gcmodel and dataset-level PFB files.

We next performed CNV calling on sample-level intensity files using QuantiSNP v2.2. We used the provided config file 'levels-hd.dat' and the provided params file 'params.dat' as input, along with developer-produced reference files for human reference genome build 37. Since QuantiSNP takes longer to run than PennCNV, we produced 'mock' Illumina FinalReport-styled files directly readable by QuantiSNP for batches of 100 samples, and parallelized the running of QuantiSNP jobs across batches to decrease overall runtime.

CNV callsets used for analysis were produced by intersecting PennCNV and QuantiSNP callsets per sample at the basepair level. Individual bases were classified as being members of a copy number variant if both were part of a CNV call in PennCNV and QuantiSNP, and if the copy number aberrations were concordant (both gains, or both losses). Contiguous segments in individual samples were combined into one single CNV event using the function 'combineseg' within the command 'clean_cnv.pl', combining segments where <20% of the total markers were found between two CNV calls. This function was called iteratively until a run resulted in no additional segment joining, yielding what we termed the 'raw' callset.

B allele frequency validation procedure

The B allele frequency (BAF) validation procedure follows a workflow utilized in Sánchez *et al.* (https://biopsych.dk/iPsychCNV/, [1]). The workflow was initially designed for particularly noisy CNV calls, and while our dataset is not nearly as noisy as that described in Sánchez *et al.* we still found value in using this procedure to remove calls that were likely false positives due to the distribution of BAFs within call loci. For this procedure, for a given CNV called within a single sample, we first extracted the B allele frequencies for all probes within the callsite, of which there should be at least 15. We first sorted the BAF values within the call site by increasing value. The

distribution of BAF values in the subsequent distribution range from 0 to 1, and are predictable given the underlying true copy state of the locus in question. For a typical autosomal locus with a copy state of 2, we expect to see 3 peaks of BAF values centered at (0, 0.5, 1). For a deletion locus with a copy state of 1, we expect to see peaks at (0, 1). For duplications with states of 3 and 4, we expect to see peaks at (0, 0.33, 0.66, 1) and (0, 0.25, 0.5, 0.75, 1) respectively. For each of these potential true copy states (c) for the CNV locus in question (1, 2, 3, 4) we perform K-means clustering where we set $k = c + 1$ (2, 3, 4, 5), and calculate a silhouette score for the fit of each k parameter to the BAF distribution within the CNV call locus. The most likely copy state based on the BAF distribution is defined as that with the highest silhouette score for the corresponding k value. We kept deletion calls where the optimal k was 2 (corresponding to $c = 1$) and we kept duplication calls where the optimal k was either 4 or 5 (corresponding to $c = 3$ and $c = 4$ respectively).

Tests for loci harboring CNV calls that drive genomic inflation

Before conducting formal association tests, we sought to determine if the CNV loci aggregated in a manner indicating bias in case/control CNV calling. To do this, we formed clusters of overlapping CNV loci and turned them into test units, conducting case/control tests of burden across each one. We did this for small CNVs (30kb-100kb) first, as a final CNV quality control step. Clusters of overlapping small CNVs found far more often in case or control groups are more likely to be due to technical differences rather than biological differences. For small CNVs, we noted p-value inflation for deletions specifically (lambda=1.21). We took the subset of loci in these tests that had an uncorrected p-value < 0.05, of which there were 3 separate loci (chr3:4182456-4325025, chr6:162713427-162868012, chr8:30544723-30594942). We removed all CNVs 30-100kb in size that overlapped one of these 3 loci (19 total), leaving behind a total of 3446 calls for our analysis. Across these 3446 calls we found no substantial evidence for test statistic inflation for deletions (lambda=1.02) or duplications (lambda=1.01).

Power analysis

We ran a power analysis using the software package CaTS v0.0.1 (https://csg.sph.umich.edu/abecasis/cats/, [2]). We first used this software to determine the range of CNV frequencies and effect sizes we could expect to identify across a range of power estimates for our case/control cohort. We defined a dominant model in our analyses, with OCD prevalence of 0.01, a frequency spectrum from 0.0001 to 0.01, and a relative risk spectrum from 1.1 to 100. We set the multiple test correction threshold alpha=2.5×10^{-6} (conservative for CNV study, but standard for exome sequencing study and a Bonferroni adjustment for 20,000 genes). For each CNV frequency value, we incrementally increased our relative risk estimate until our subsequent power calculated passed the power threshold being tested. The power curves are shown below:

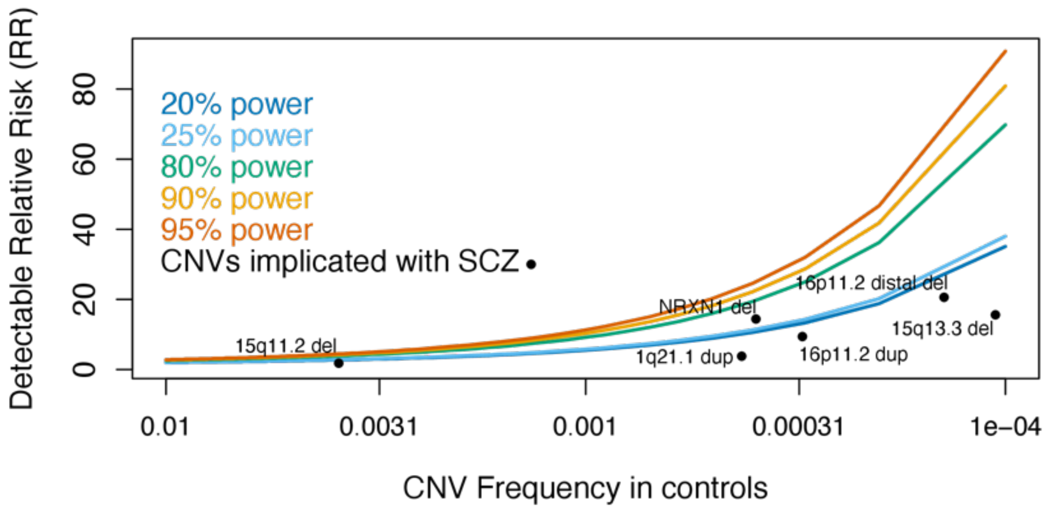

We next plotted power curves for CNVs we might be well-powered to identify within our study (defined here as 80% power) relative to a theoretical sample size from a substantially larger study focused on Schizophrenia that successfully identified multiple risk CNV loci [3]. The frequencies and effect sizes from CNVs implicated in the Schizophrenia CNV studies are shown, along with labels for risk CNV identities. For all curves plotted here we utilized the same parameter values that were previously described. We see that for many of these risk CNVs, sample sizes in the range of the Schizophrenia study are required to get adequate power for detection:

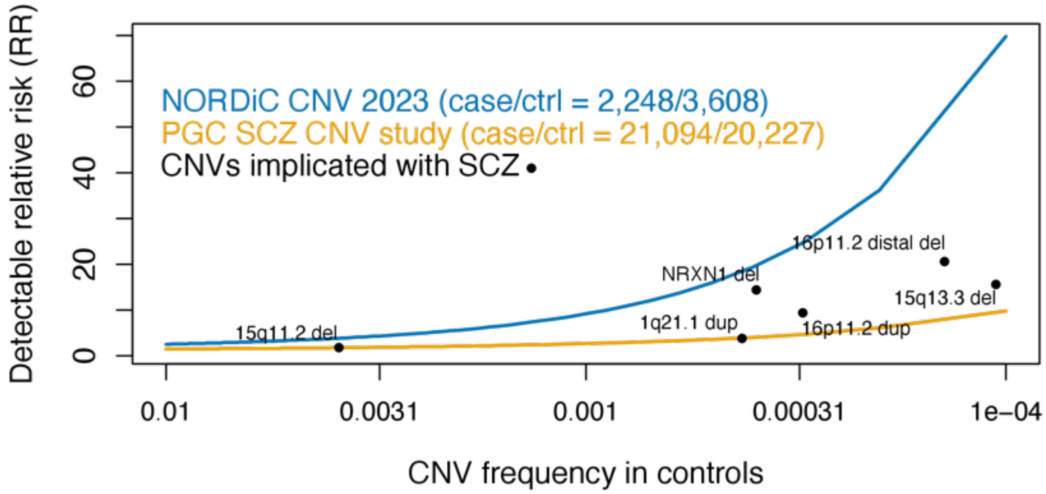

We conducted one more power analysis comparing CNVs we're well-powered to detect relative to a much larger CNV study. We relaxed the multiple test correction to alpha=9.43×10^{-4} (Bonferroni correction for 53 known neurodevelopmental CNVs from Kendall *et al.*, [4]). We see below that even with a relaxed multiple testing threshold, only considering known neurodevelopmental CNVs, that we are still underpowered to detect risk CNVs at the frequencies and effect sizes described in the Schizophrenia CNV study:

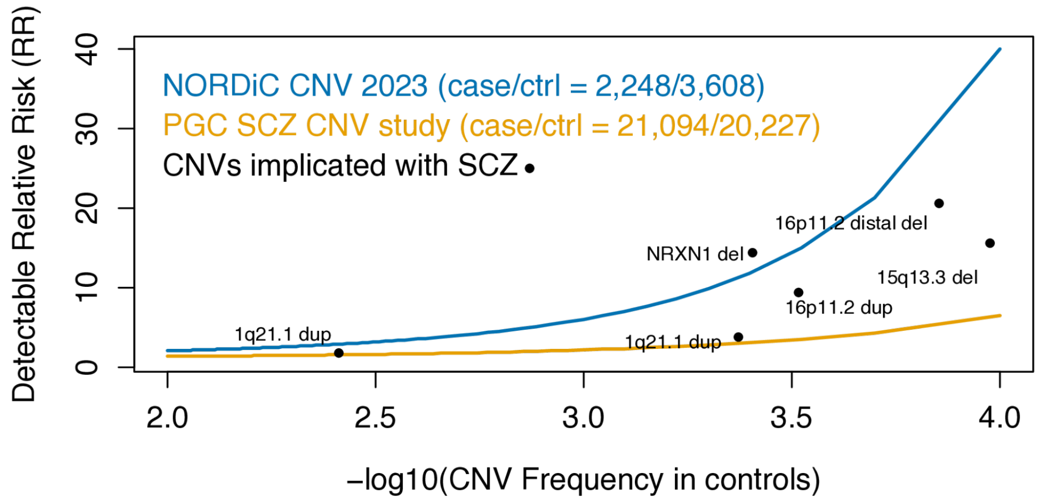

Association tests between CNV burden, country-of-origin and sex

We constructed a series of 3 different pairwise comparisons to determine if there was evidence for a difference in deleterious CNV burden in the Sweden case cohort versus the Norway case

cohort. The first and second were comparisons of Swedish OCD cases versus Swedish controls specifically, and Norwegian OCD cases versus Norwegian controls. These comparisons were meant mainly to verify that within these comparisons we still saw evidence for an excess of deleterious CNVs in cases relative to controls in both Sweden and Norway, separately. The third and most important comparison was Swedish OCD cases versus Norwegian OCD cases. In all comparisons we utilized a simplified logistic regression model, where in the first two comparisons, case status is the outcome, and in the third comparison, Norwegian origin (as opposed to Swedish origin) was the outcome. For all three sets of comparisons we utilized sex and the total number of non-genic CNVs per sample as covariates. We tested 5 categories of CNV burden, 1) CNVs where the maximum overlapping gene pLI is less than 0.5 (a negative control), 2) CNVs where the maximum pLI is greater than 0.5, 3) Neurodevelopmental CNVs as defined in Kendall *et al.* 2019 [4], 4) CNVs overlapping a Neurodevelopmental gene defined in Fu *et al.* [5], and 5) CNVs > 1 megabase in size. In plotting the results, for greater visibility, we cut off 95% confidence intervals that extended beyond case/control odds ratio=8.

We utilized this same approach for assessing CNV burden in male and female samples. All 5 of the same categories of CNV burden previously described were used, and instead of splitting samples into groups based on country-of-origin (Sweden, Norway) we split samples into groups based on biological sex as determined from genotype array data (male, female). The same linear regression model and covariates were used, only here, in the first two models (male-only, female-only), case status is the outcome, and in the third model (cases only), female sex was the outcome.

Pathway enrichment analysis

We performed a series of pathway enrichment analyses in order to see in an unbiased manner if there were particular biological processes or tissue expression profiles with a significant difference in the number of overlapping CNV-impacted genes in cases versus controls. We

focused on all CNVs (deletions and duplications combined), rather than deletions only or duplications only, since our other results show that while the deletion signal is stronger, there is detectable duplication signal as well, and that given the low frequency of CNVs and the small sample size, combining deletion and duplication signal should help with power.

We extracted genesets for inclusion in our pathway enrichment analyses from two different sources. The first was the Gene Ontology (GO) consortium, which categorizes known genes by molecular functions and cellular locations, assigns IDs to these groupings, and has listings of ontological relationships between these genesets. We utilized the basic version of GO (go-basic.obo, version 2024-04-24) along with corresponding human annotations (goa_human.gaf.gz, generated 2024-04-25). We were able to derive a total of 18,650 genesets from these files. The second was a collection of genesets described in Bryois *et al.* ([6], files from https://github.com/jbryois/scRNA_disease) that each represent the expression profile of tissues from the Genotype-Tissue Expression project (version 8, 37 tissues total). As a positive control, we included genes with pLI>0.5 (4543 genes total) which in our analysis have a significant elevation of overlapping CNVs in cases relative to controls. As a negative control, we included genes with a probability of null impact from loss of function greater than 0.5 (pNull>0.5), since this allowed us to get a set of genes the same size as our positive control (4540 genes total). We only considered the subset of these genesets where across the total case/control cohort, at least 5 separate instances of a gene being impacted in a sample were observed. Placing this requirement left us with 2226 tests to perform in total.

In our association tests, for each geneset, we tested the null hypothesis of the per-sample number of CNV-impacted genes within the geneset in cases versus controls being equal. We did this using a logistic regression model, with the same covariates described in analyses from the same text, along with an additional covariate for the total number of CNV-impacted genes in

each sample. We corrected for multiple tests using the Benjamini-Hochberg procedure and considered a result as significant if the FDR-adjusted Q-value was less than 0.1.

Given that the positive control pLI>0.5 was the only geneset to survive multiple test correction, we considered a second round of association tests where we specifically subset only on genes with pLI>0.5. We used the same logistic regression model described above, but the covariate for the total number of CNV-impacted genes in each sample was changed to total number of CNV-impacted genes per sample with pLI>0.5, to control for the baseline elevation of CNVs in these intolerant genes. As before, we considered results significant if the corresponding FDR-adjusted Q-value is less than 0.1.

References

1. Calle Sánchez X, Helenius D, Bybjerg-Grauholm J, Pedersen C, Hougaard DM, Børglum AD, et al. Comparing Copy Number Variations in a Danish Case Cohort of Individuals With Psychiatric Disorders. JAMA Psychiatry. 2022;79:59–69.
2. Skol AD, Scott LJ, Abecasis GR, Boehnke M. Joint analysis is more efficient than replication-based analysis for two-stage genome-wide association studies. Nat Genet. 2006;38:209–213.
3. Marshall CR, Howrigan DP, Merico D, Thiruvahindrapuram B, Wu W, Greer DS, et al. Contribution of copy number variants to schizophrenia from a genome-wide study of 41,321 subjects. Nat Genet. 2017;49:27–35.
4. Kendall KM, Rees E, Bracher-Smith M, Legge S, Riglin L, Zammit S, et al. Association of Rare Copy Number Variants With Risk of Depression. JAMA Psychiatry. 2019;76:818–825.
5. Fu JM, Satterstrom FK, Peng M, Brand H, Collins RL, Dong S, et al. Rare coding variation provides insight into the genetic architecture and phenotypic context of autism. Nat Genet. 2022;54:1320–1331.
6. Bryois J, Skene NG, Hansen TF, Kogelman LJA, Watson HJ, Liu Z, et al. Genetic identification of cell types underlying brain complex traits yields insights into the etiology of Parkinson’s disease. Nat Genet. 2020;52:482–493.
